# Supplementary material for: Early Endosomal Vps34-Derived Phosphatidylinositol-3-Phosphate Is Indispensable for the Biogenesis of the Endosomal Recycling Compartment
Source: Cells. 2022 Mar 11;11(6):962. doi: 10.3390/cells11060962 (PMC8946653; doi:10.3390/cells11060962)
Supplement: Supplementary file 1 [file cells-11-00962-s001.zip › cells-1581976-supplementary.pdf]

Supplementary Material

Supplementary Figures

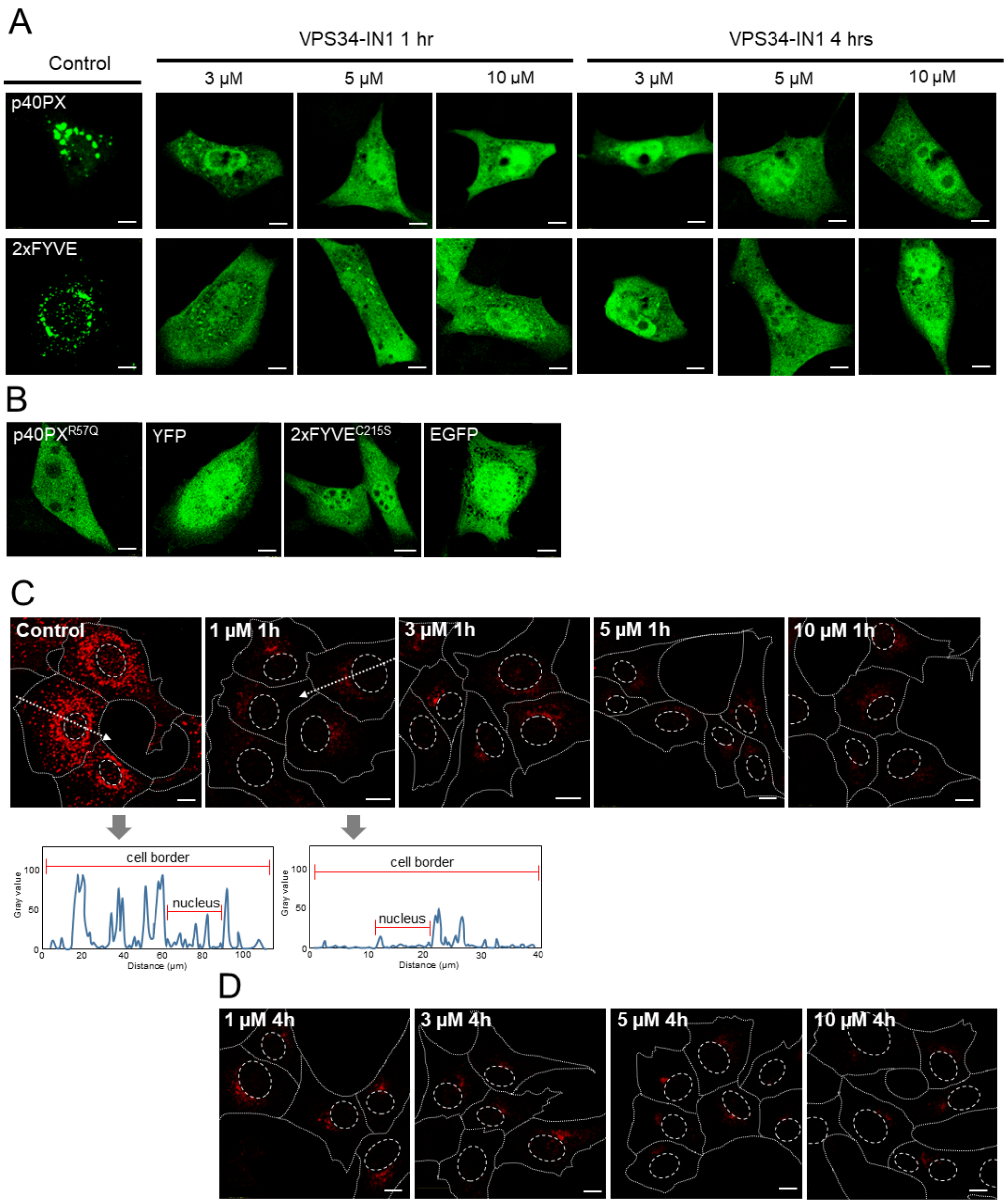

Figure S1. **Pharmacological inhibition of Vps34 rapidly dissociates PI3P-binding proteins from EEs** (*related to Fig. 1C*). (**A**) *Dissociation of fluorescent PI3P-binding domains***.** Images of Balb 3T3 cells transfected with YFP-PX$_{p40phox}$ MSCV (p40PX) or EGFP-2xFYVE$_{Hrs}$ MSCV (2xFYVE) and after 48 hrs treated with various concentrations of VPS34-IN1 for 1 and 4 hrs. (**B**) Images of cells transfected either with YFP-PX$_{P40phox}^{R57Q}$ MSCV (p40PX^{R57Q}), YFP MSCV, EGFP-2xFYVE$_{Hrs}^{C125S}$ MSCV (2xFYVE^{C125S}), or EGFP MSCV after 48 hrs post-transfection. (**C-D**) *Dissociation of EEA1*. Balb 3T3 cells were treated with various concentrations of IN1 for 1 and 4 hrs, fixed, permeabilized, and stained with mAb against EEA1 and anti-chicken IgG AF^{555}. Colocalization analysis was performed by plotting fluorescence intensity profiles along white dashed lines. Cell borders are indicated by fine dotted lines and nuclei by fine dashed lines. Bars, 10 μm.

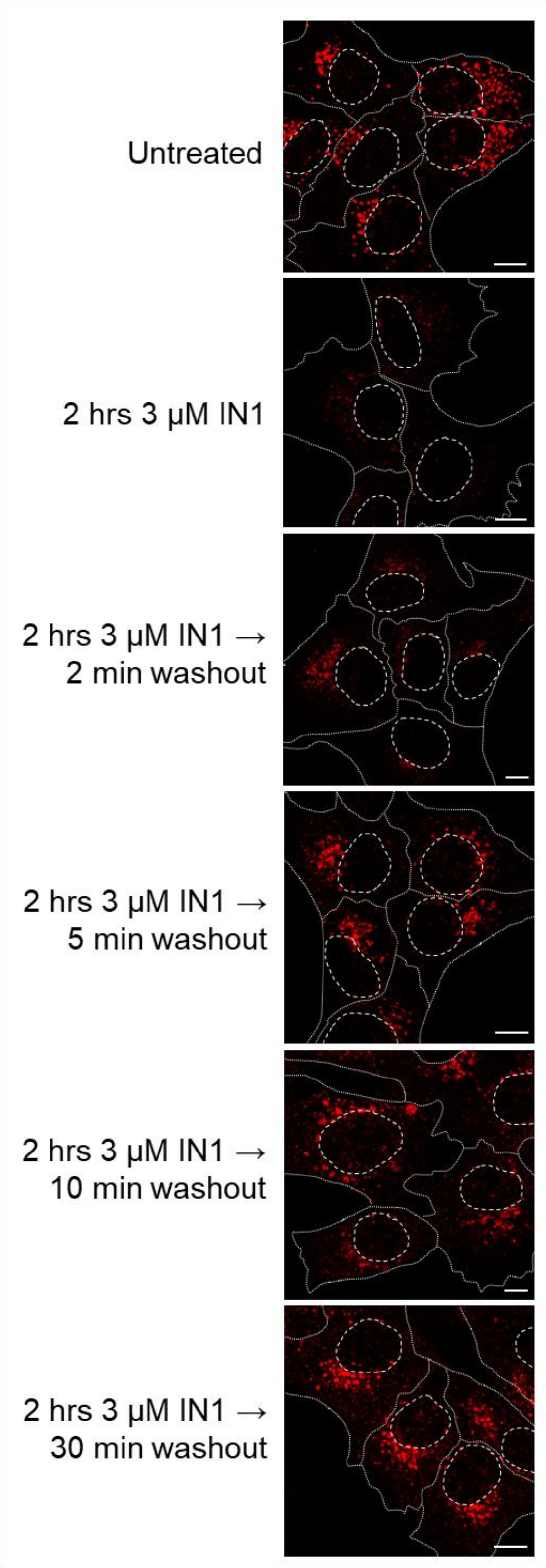

Figure S2. **Rapid restoration of endosome-associated EEA1 after VPS34-IN1 washout (***related to Fig. 1E***)**. Subcellular distribution of EEA1 in untreated and VPS34-IN1 (IN1)-treated Balb 3T3 cells after IN1 washout. Cell borders are indicated by fine dotted lines and nuclei by fine dashed lines. Bars, 10 μm.

Blots probed for EEA1 and β-actin

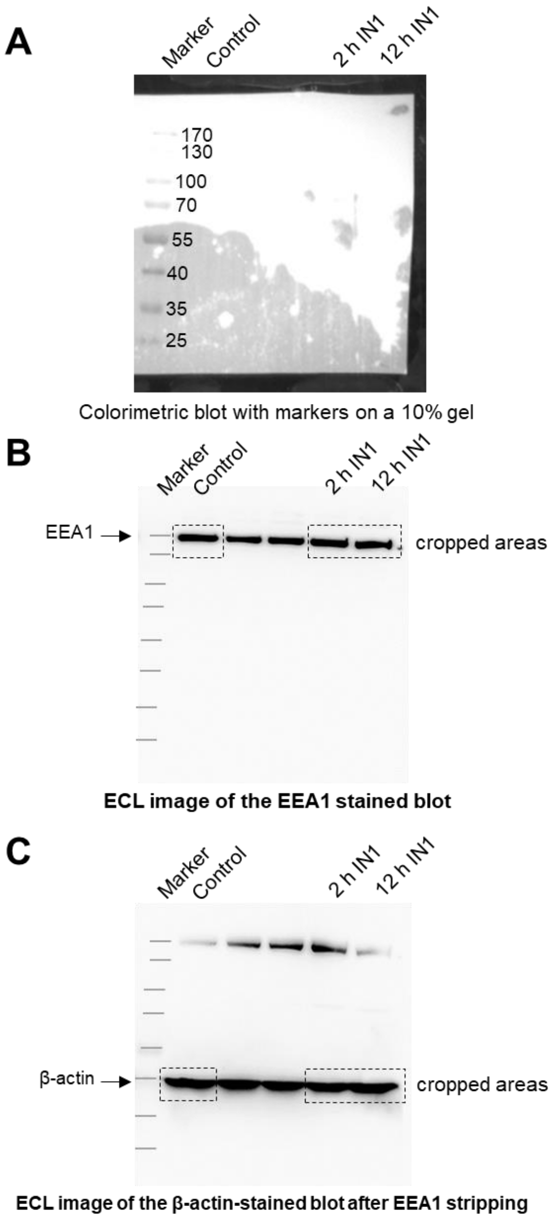

Figure S3. **Original raw blots and unprocessed ECL images of EEA1 and β-actin used as a representative western blot in Fig. 1F of the manuscript.** The original membrane with various samples (**A**), including samples presented in Fig. 1F (labeled lines), used for staining against EEA1 (**B**), and β-actin (**C**) after stripping off the anti-EEA1. The expected molecular weight of EEA1 was 170 kDa. For staining of EEA1 we used rabbit monoclonal antibody (C45B10 #3288 - Cell signaling 1:1,000) followed by anti-rabbit IgG-POD (Jackson 1:50,000). For staining of β-actin we used mouse monoclonal antibody (Millipore, Cat.No. MAB1501; 1:80,000) followed by anti-mouse IgG-POD (Jackson 1:50,000). The reaction was visualized using SignalFire (TM) Plus ECL Reagent (Cell Signaling, 12630S, LOT No:6). The molecular weight markers used in these experiments are PageRuler Prestained Protein Ladder (Thermo Scientific, Product: 26616, LOT: 00515508). For blotting, we used PVDF membranes (Merck Millipore, size: 0.45um, Immobilon-P Transfer Membranes CAT: IPVH00010, LOT: R6PA1239C). M, marker line. Densitometric analysis of western blot signals shown in A, analyzed by Image J 1.53 software and normalized to actin signal used as a loading control. We first calculated the normalization factor for every lane according to the formula: *Lane normalization factor = Observed signal of actin for every lane/Highest observed signal of actin* for the blot. Following that, *Normalized experimental signals* were calculated as the *Observed experimental signal/Lane normalization factor ratio*.

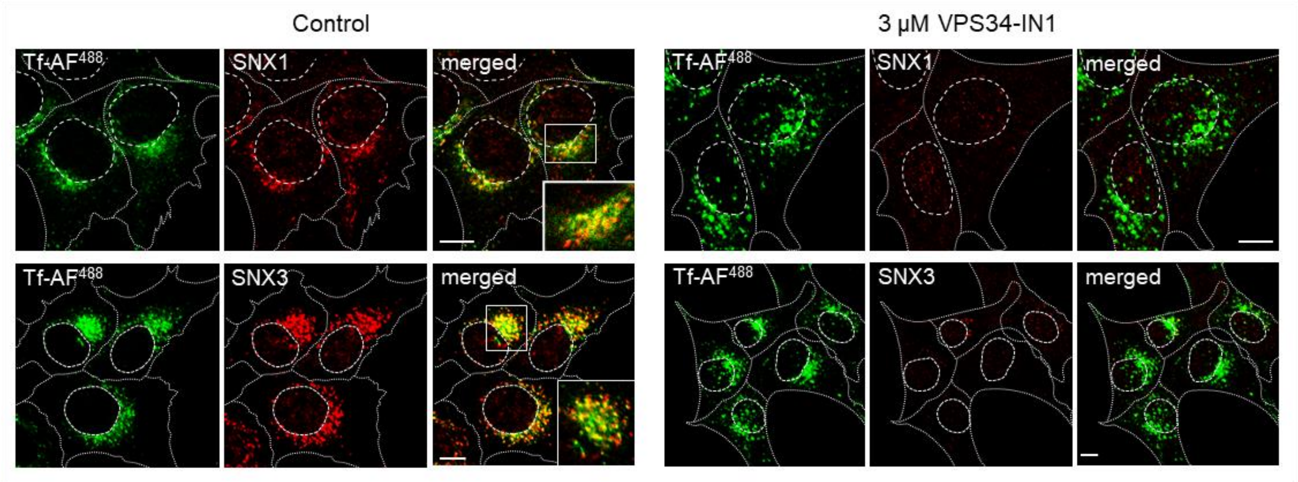

Figure S4. **Dissociation of early endosomal sorting nexins (SNX) after PI3P depletion (***related to Fig. 1H***)**. Representative images (N=8-12) of internalized Tf-AF^{488} and SNXs (SNX1 and SNX3) in untreated and VPS34-IN1-treated Balb 3T3 cells. In untreated cells, Tf-AF^{488} (50 µg/ml) was added directly to the tissue culture medium, whereas in 3µM IN1-treated cells Tf-AF^{488} was added 15 min after IN1, without IN1 washout, and incubated for 45 min. The antibodies used are listed in Table S1, and each marker is described in Table S2. Insets present zoomed area box. Cell borders are indicated by fine dotted lines and nuclei by fine dashed lines. Bars, 10 µm.

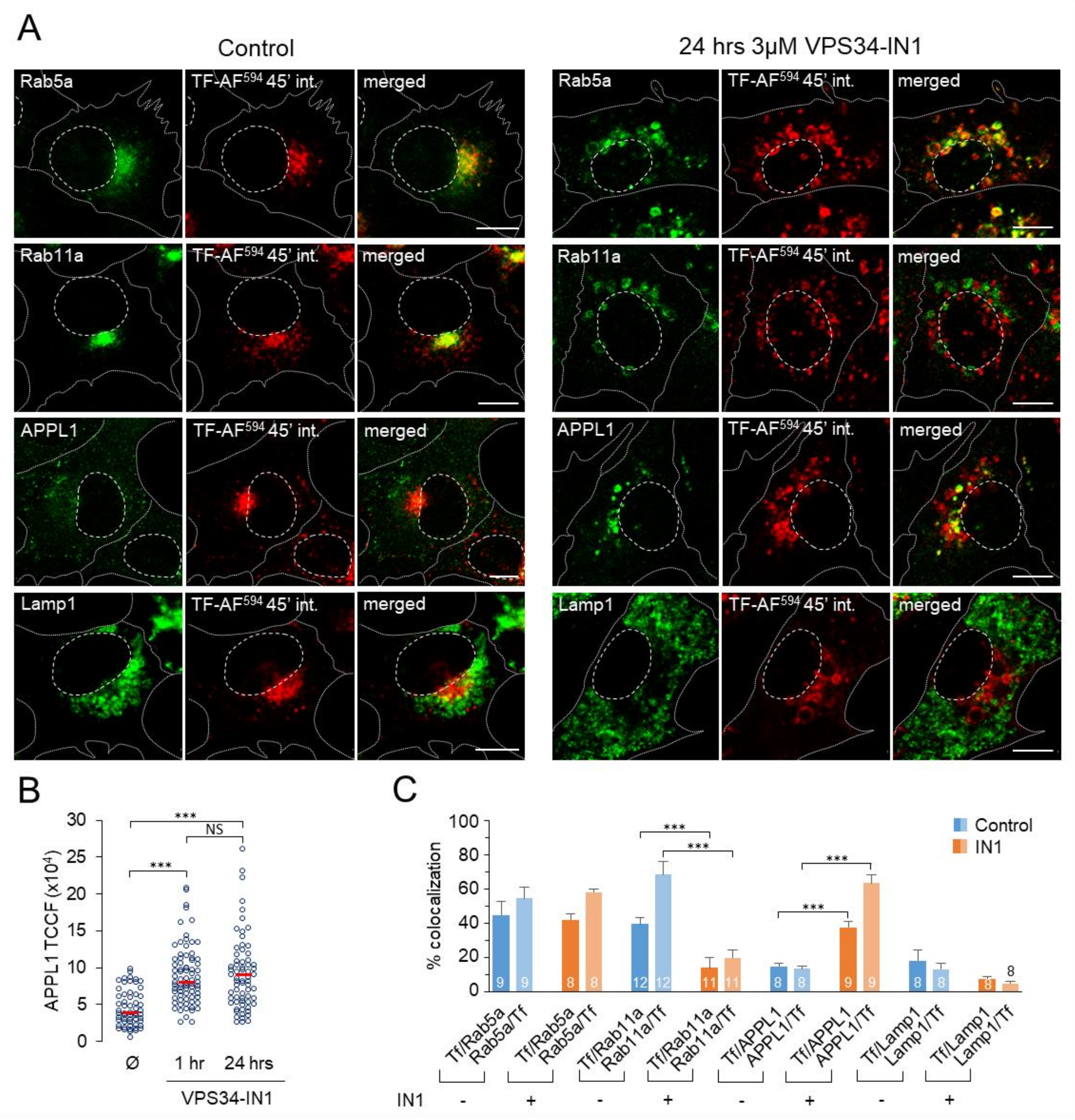

Figure S5. **Colocalization analysis of internalized Tf with membranous organelle markers in long-term PI3P-depleted cells (***related to Fig. 3***).** (**A**) Colocalization of internalized Tf-AF^{594} (45 min) with Rab5a, Rab11a, APPL1, and Lamp1 in control and 24 hrs IN1-treated (3 μM) Balb 3T3 cells. Shown are the representative confocal images (N=8). Cell borders are indicated by fine dotted lines and nuclei by fine dashed lines. Bars, 10 μm. (**B**) Quantification of APPL1 signal by ImageJ analysis. Dots represent the Total Corrected Cell Fluorescence (TCCF) of individual cells in a representative experiment (N=3), and red bars the median value (**C**). The 3D colocalization of Tf with organelle markers and organelle markers with Tf in control and 24 hrs IN1-treated cells based on M1/M2 coefficients of pixel overlap measured across the Costes-algorithm thresholded z-stacks of confocal images. Data represent mean±SEM per cell (number of cells indicated within bars).

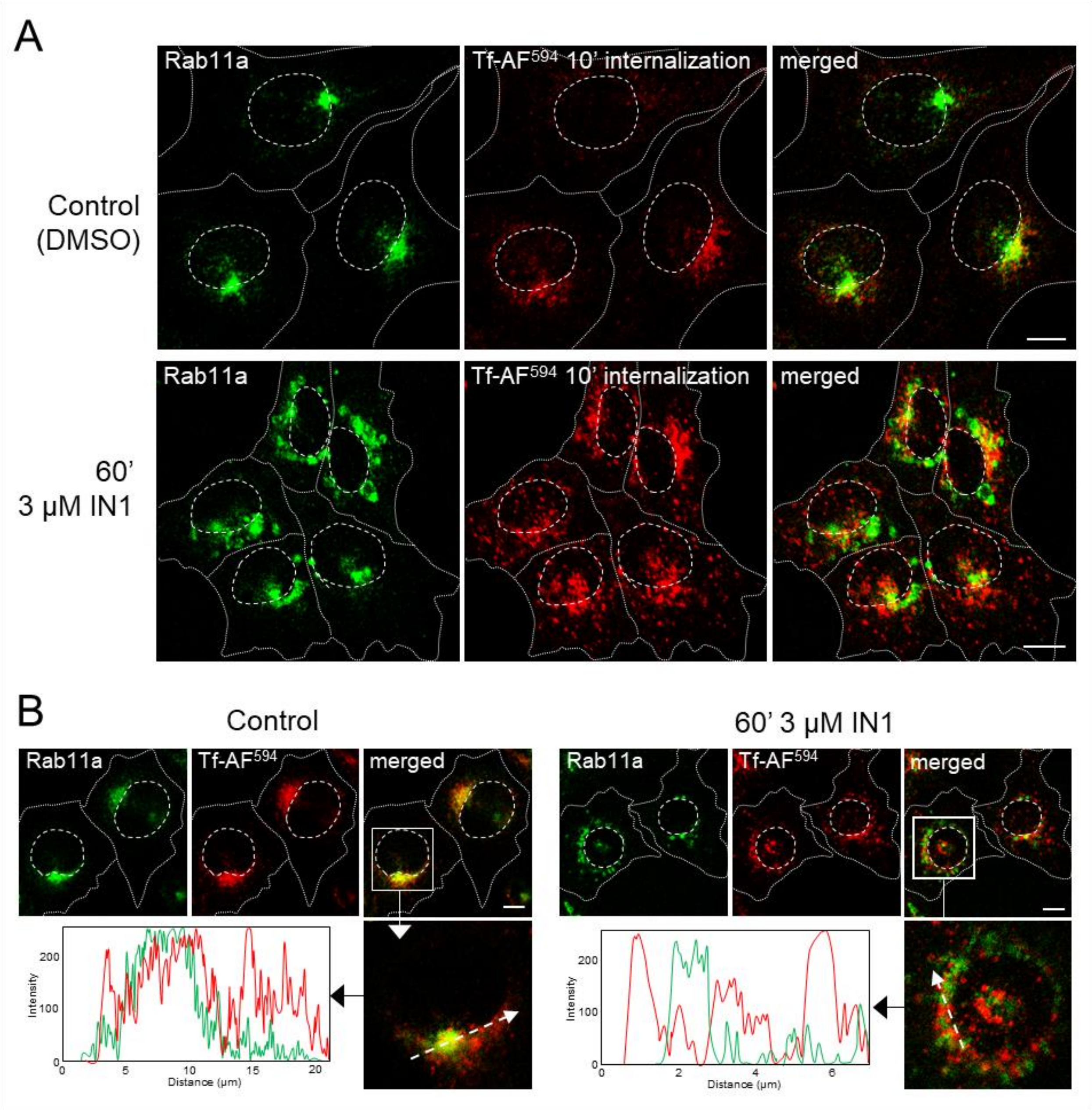

Figure S6. **Colocalization analysis of internalized Tf with Rab11a in short-term PI3P-depleted cells (***related to Fig. 3***).** (**A**) Representative (N=13) images of internalized Tf-AF^{594} (10 min uptake) and Rab11a in control and IN1-treated Balb 3T3 cells. Cell borders are indicated by fine dotted lines and nuclei by fine dashed lines. Bars, 10 µm. (**B**) Representative (N=25) focal-plane images of internalized Tf-AF^{594} (45 min uptake) and Rab11a in control and 60 min IN1-treated cells. Inserts present zoomed area box. Colocalization analysis was performed by plotting fluorescence intensity profiles along white dashed lines. Cell borders are indicated by fine dotted lines and nuclei by fine dashed lines. Bars, 10 µm.

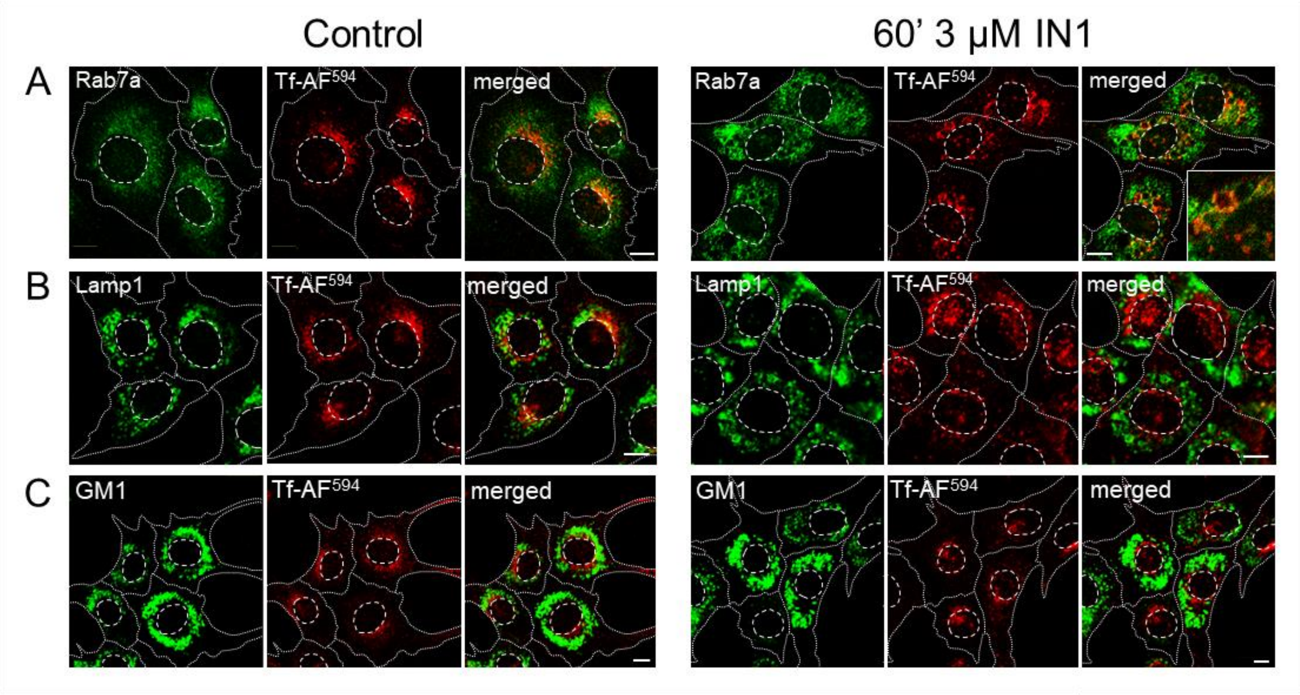

Figure S7. **Subcellular distribution of internalized Tf relative to mature late endosomes in short-term PI3P-depleted cells** (*related to Fig. 3*). Representative (N=8-12) images of internalized Tf-AF^{594} (45 min uptake) and EE internal membrane budding markers and steady-state LE markers in control and IN1-treated Balb 3T3 cells. Antibody reagents used are listed in Table S1, and each marker is described in Table S2. Cell borders are indicated by fine dotted lines and nuclei by fine dashed lines. Bars, 10 µm.

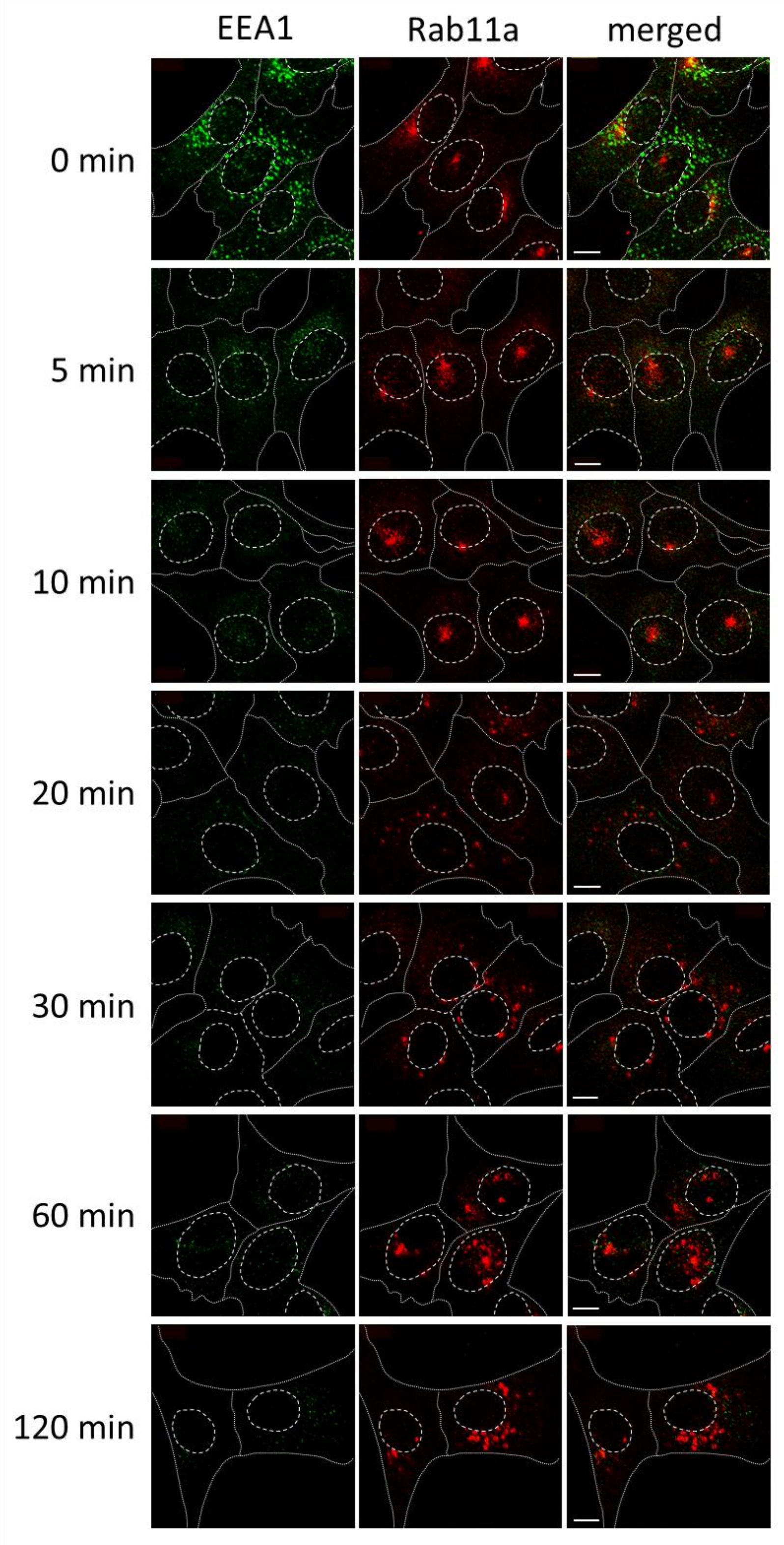

Figure S8. **Reorganization of Rab11a endosomes in PI3P depleted cells (***related to Fig. 4***).** Representative images (N=8-12) of simultaneously stained EEA1 and Rab11a in control (0 min) and 10-120 min IN1-treated Balb 3T3 cells. Cell borders are indicated by fine dotted lines and nuclei by fine dashed lines. Bars, 10 μm.

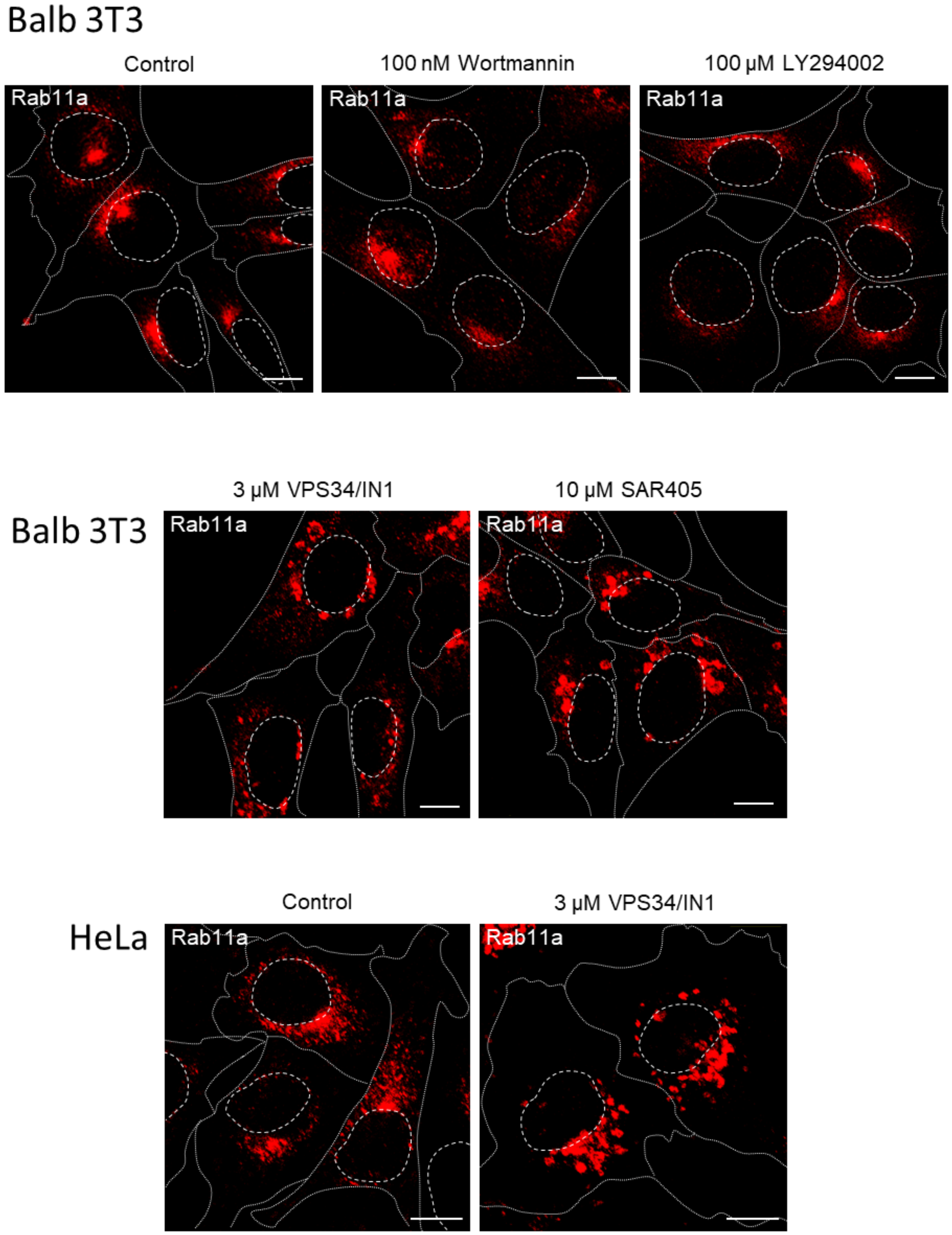

Figure S9. **Subcellular distribution of Rab11a-positive compartments in Balb 3T3 and HeLa cells treated for 120 mins with PI3K inhibitors (**related to Fig. 4**).**

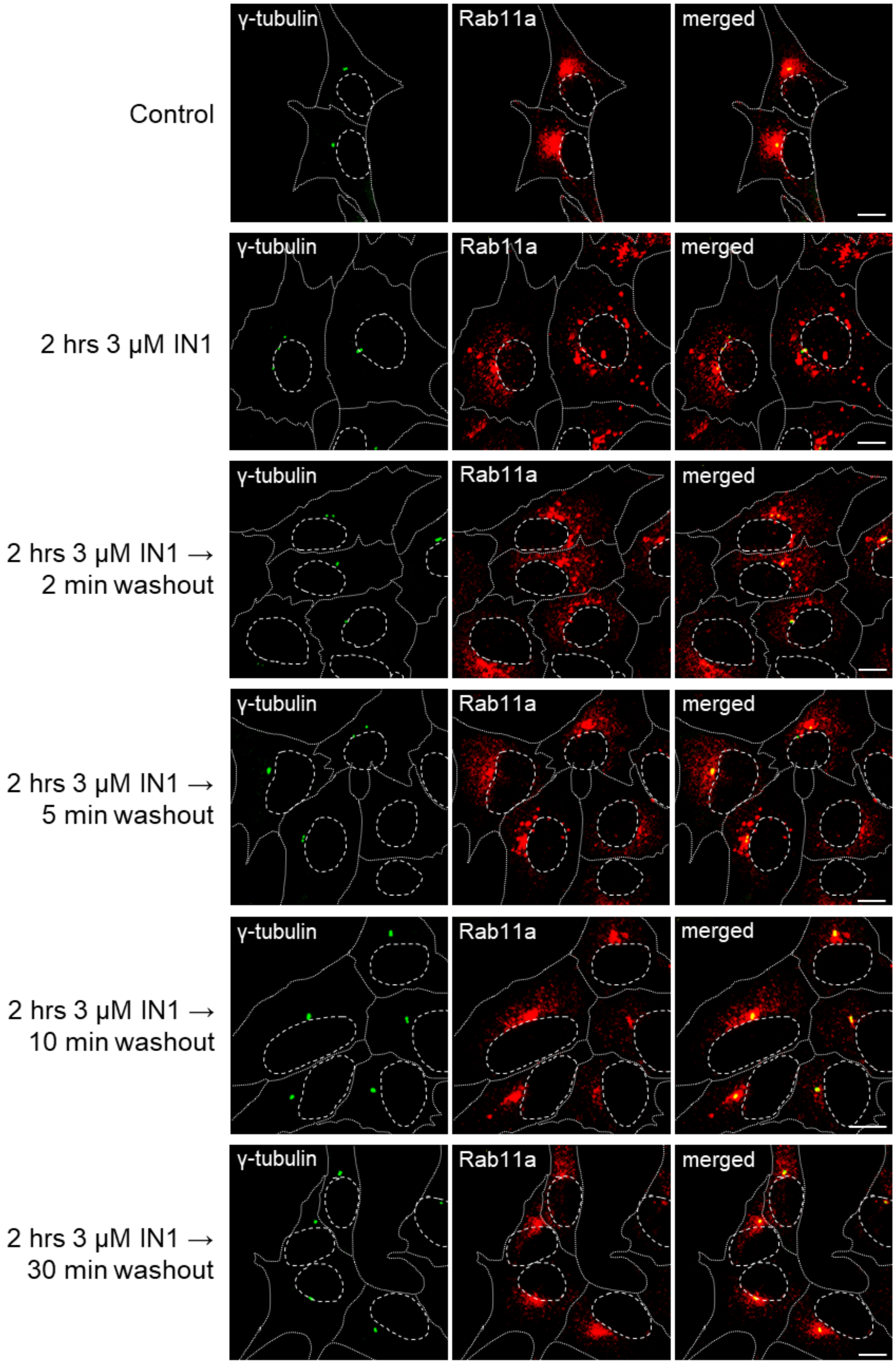

Figure S10. **Restoration of pericentriolar Rab11a accumulation after lifting the reversible Vps34 inhibition (***related to Fig. 4F and 4I***).** Representative images (N=6-8) of Rab11a distribution (red fluorescence) relative to centrosomes stained by γ-tubulin (green fluorescence) after 120 min of IN1-treatment followed by IN1 washout and incubation in IN1-free medium for 2-30 min. Cell borders are indicated by fine dotted lines and nuclei by fine dashed lines. Bars, 10 μm.

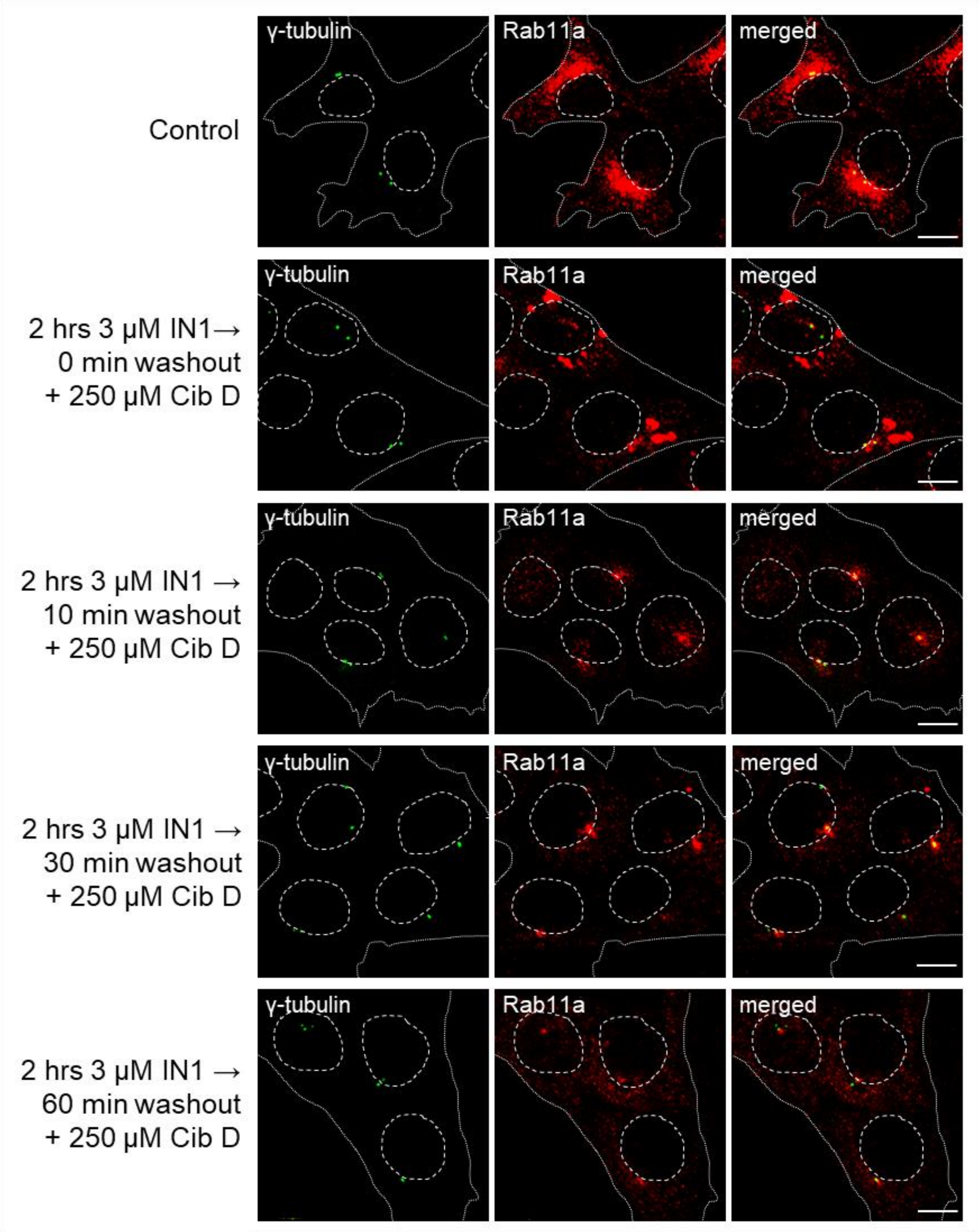

Figure S11. **Restoration of pericentriolar Rab11a accumulation after lifting the reversible Vps34 inhibition is dynein dependent (***related to Fig. 4H and 4I***)**. Representative images (N=6-8) of Rab11a distribution relative to centrosomes (γ-tubulin, green fluorescence) after 120 min of IN1-treatment followed by IN1 washout and incubation in IN1-free medium containing 250 µM Ciliobrevin D (Cib D) for 0-30 min. Cell borders are indicated by fine dotted lines and nuclei by fine dashed lines. Bars, 10 µm.

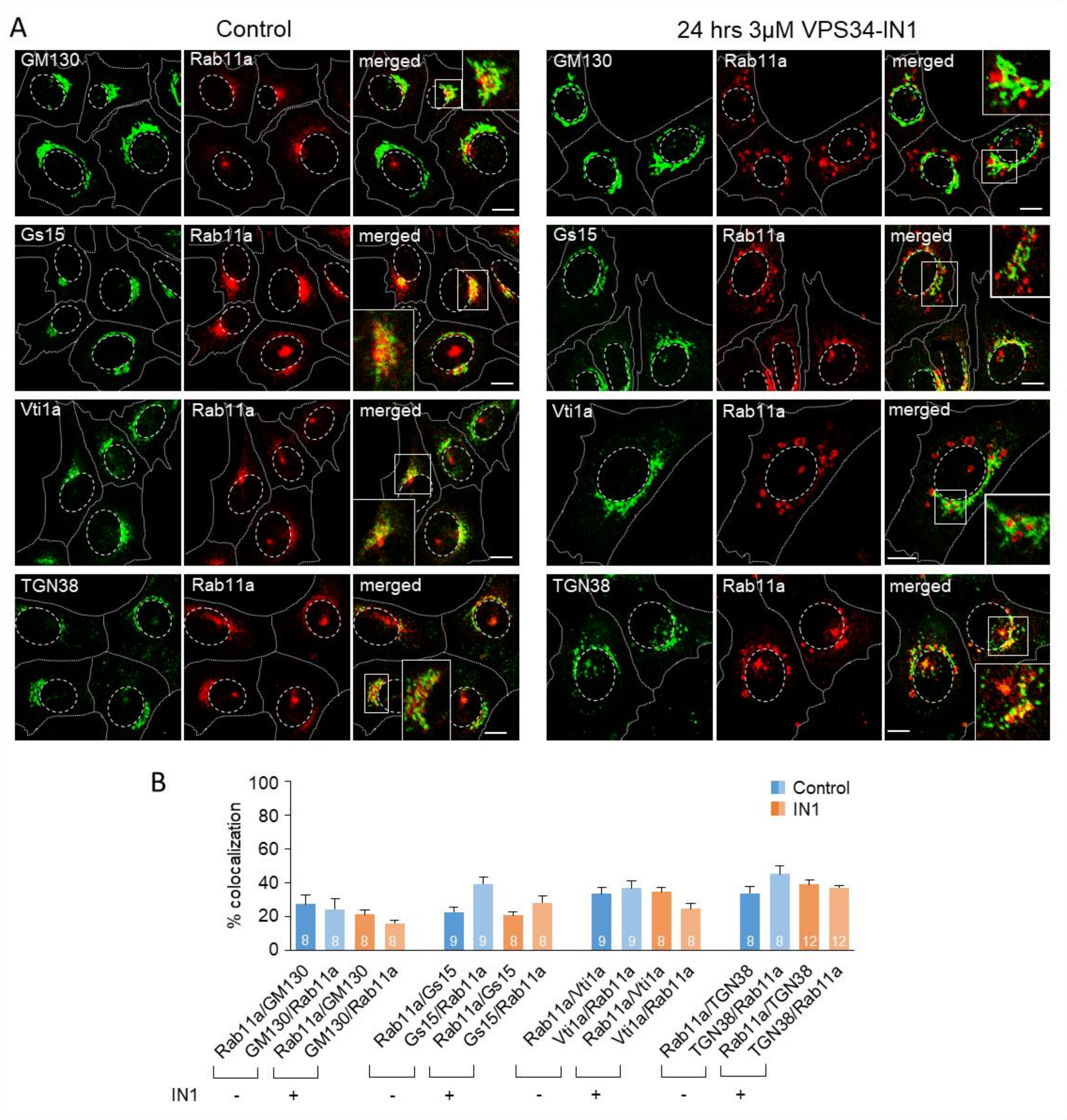

Figure S12. **Co-localization of Rab11a with the Golgi markers in long-term PI3P-depleted cells** (*related to Fig. 5*)**.** (**A**) Representative images (N=10-12) of Rab11a and cis-Golgi (GM130), medial/trans-Golgi (Gs15), and trans-Golgi network (Vti1a and TGN38) markers in control and IN1-treated Balb 3T3 cells. Insets present zoomed area box. Cell borders are indicated by fine dotted lines and nuclei by fine dashed lines. Bars, 10 μm. (**B**) The 3D colocalization of Rab11a with the Golgi markers based on M1/M2 coefficients of pixel overlap measured across the Costes-algorithm thresholded z-stacks of confocal images. Data represent mean±SEM per cell (number of cells indicated within bars).

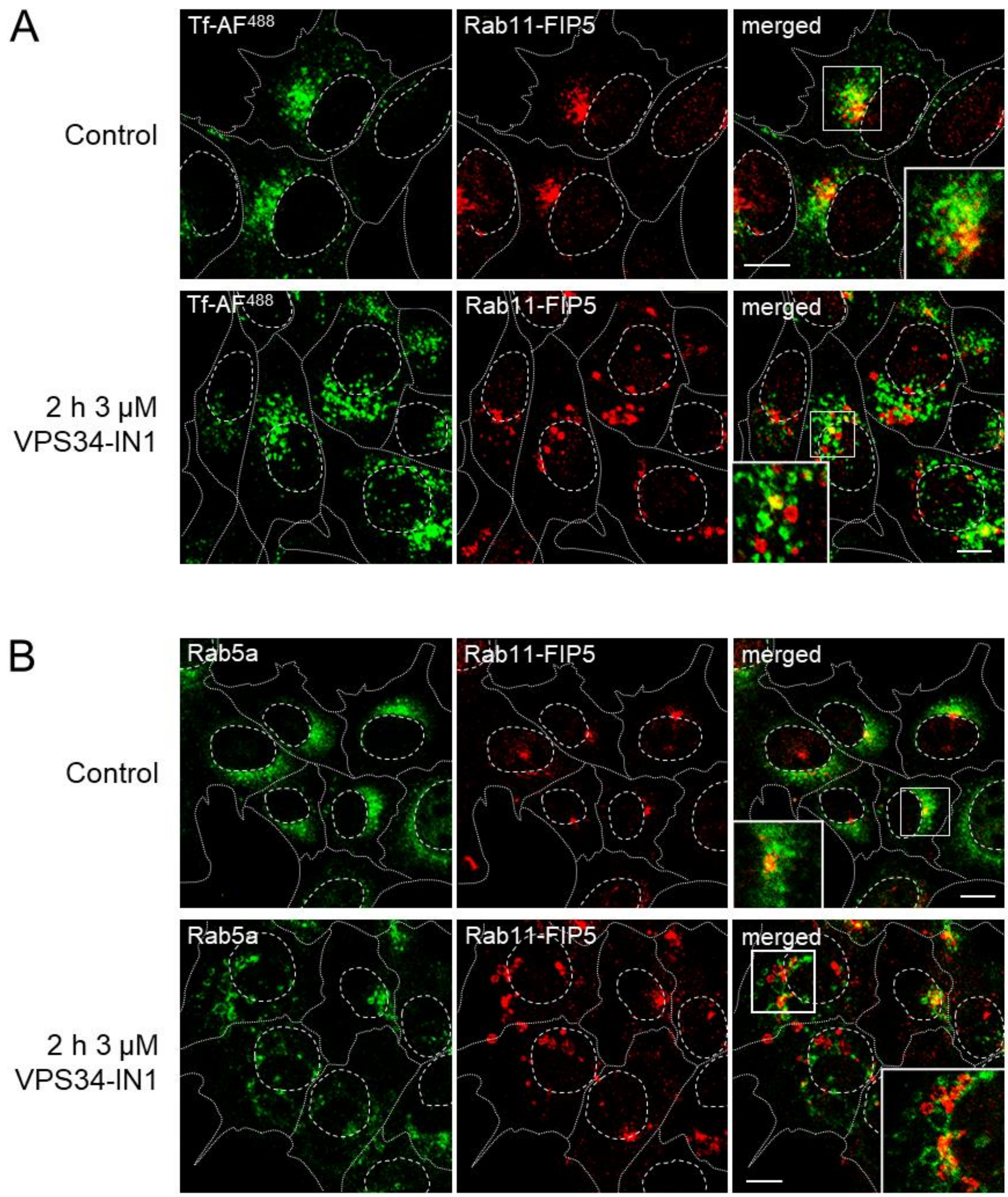

Figure S13. **Colocalization of Rab11-FIP5 with internalized Tf and Rab5a in PI3P-depleted cells (***related to Fig. 7***).** (**A**) Representative (N=8-12) images of internalized Tf-AF^{488} (45 min uptake) and Rab11-FIP5 (FIP5) in control and IN1-treated Balb 3T3 cells. (**B**) Representative (N=8-12) images of endogenous Rab5a and FIP5 in control and IN1-treated cells. The antibodies used are listed in Table S1, and each marker is described in Table S2. Insets present zoomed area box. Cell borders are indicated by fine dotted lines and nuclei by fine dashed lines. Bars, 10 µm.

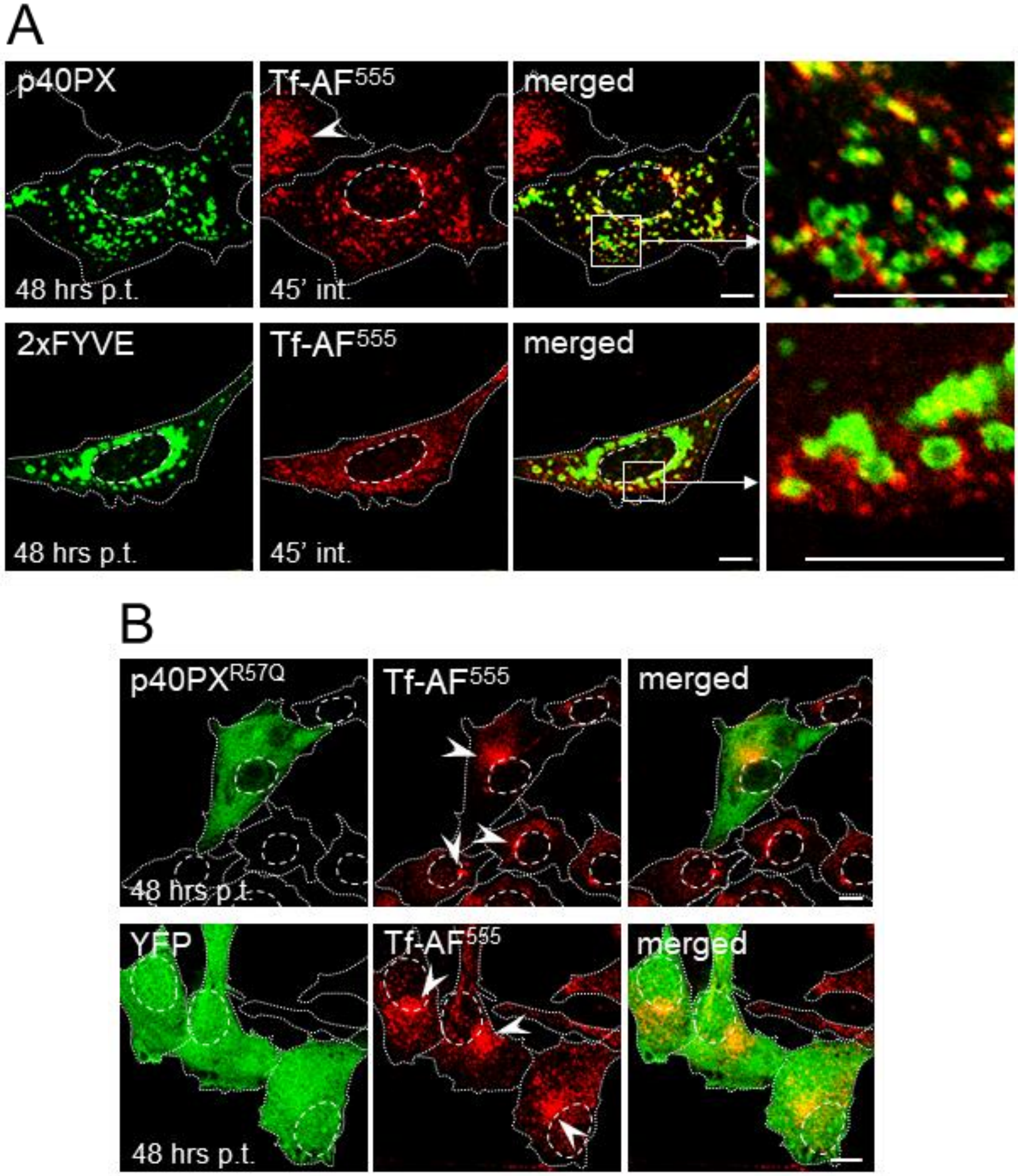

Figure S14. **Prolonged expression of PI3P-binding domains alters TfR trafficking (***related to Fig. 8***)**. Balb 3T3 cells were transfected with EGFP-2xFYVE MSCV, YFP-p40PX-MSCV, YFP-p40PX^{R57Q}-MSCV, or YFP-MSCV. 48 hrs after transfection (p.t.), the cells were incubated for 45 min with Tf-AF^{555} at 37°C and analyzed by confocal microscopy. Arrowheads point to the juxtanuclear endosomes loaded with internalized Tf-AF^{555}. Cell borders are indicated by fine dotted lines and nuclei by fine dashed lines. Bars, 10 μm.

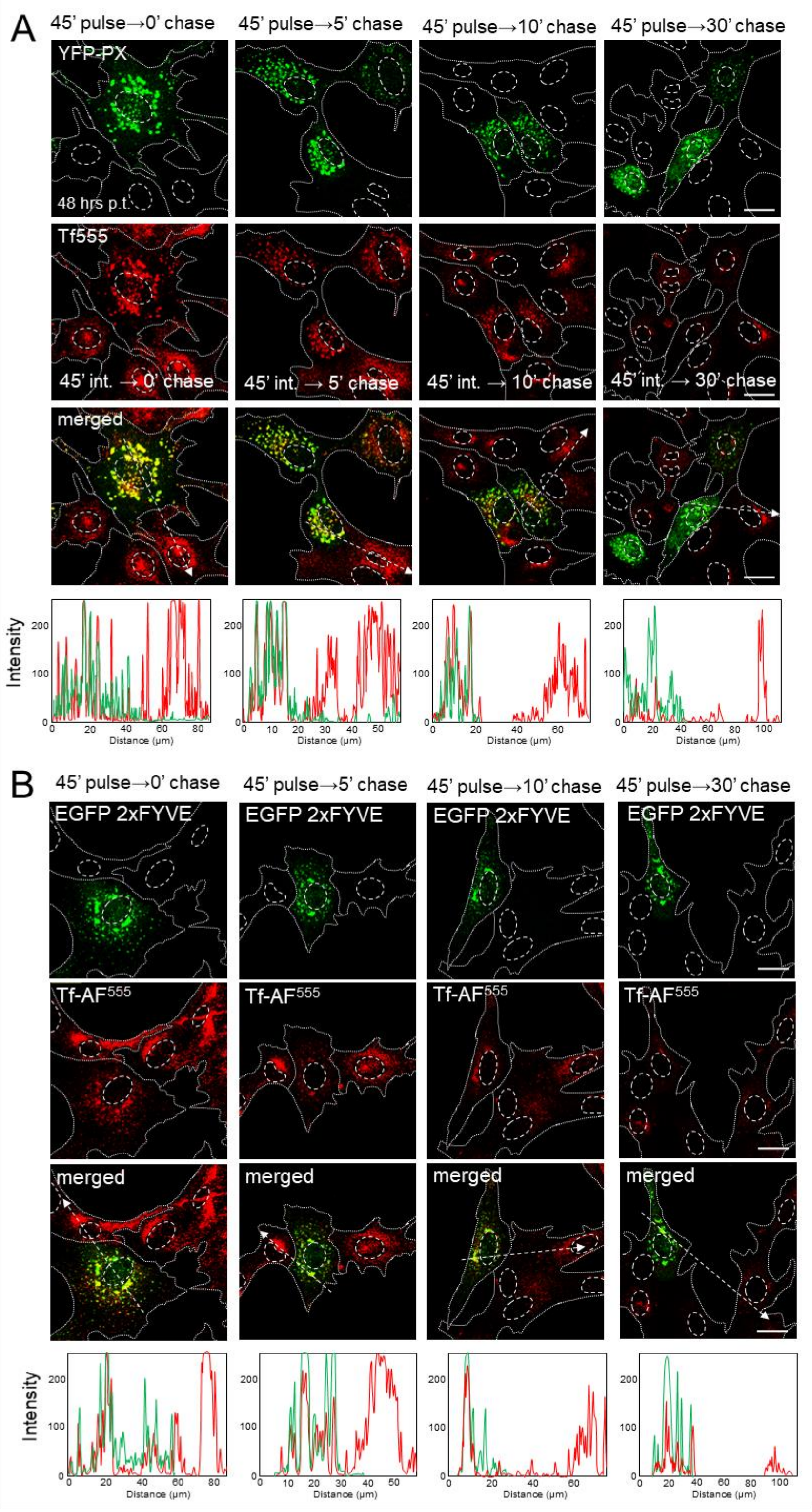

Figure S15. **Pulse-chase analysis of TfR recycling in YFP-PX and EGFP-2xFYVE transfected cells (***related to Fig. 8***)**. Balb 3T3 cells transfected for 48 hrs either with EGFP-2xFYVE-MSCV or YFP-PX-MSCV were incubated 45 min at 37°C with AF^{555}-Tf (pulse), chased 0-30 min at 37°C in medium containing unlabeled Tf, and analyzed by confocal imaging. Colocalization analysis was performed by plotting fluorescence intensity profiles along white dashed lines. Cell borders are indicated by fine dashed lines and nuclei by fine dotted lines. Bars, 10 µm.

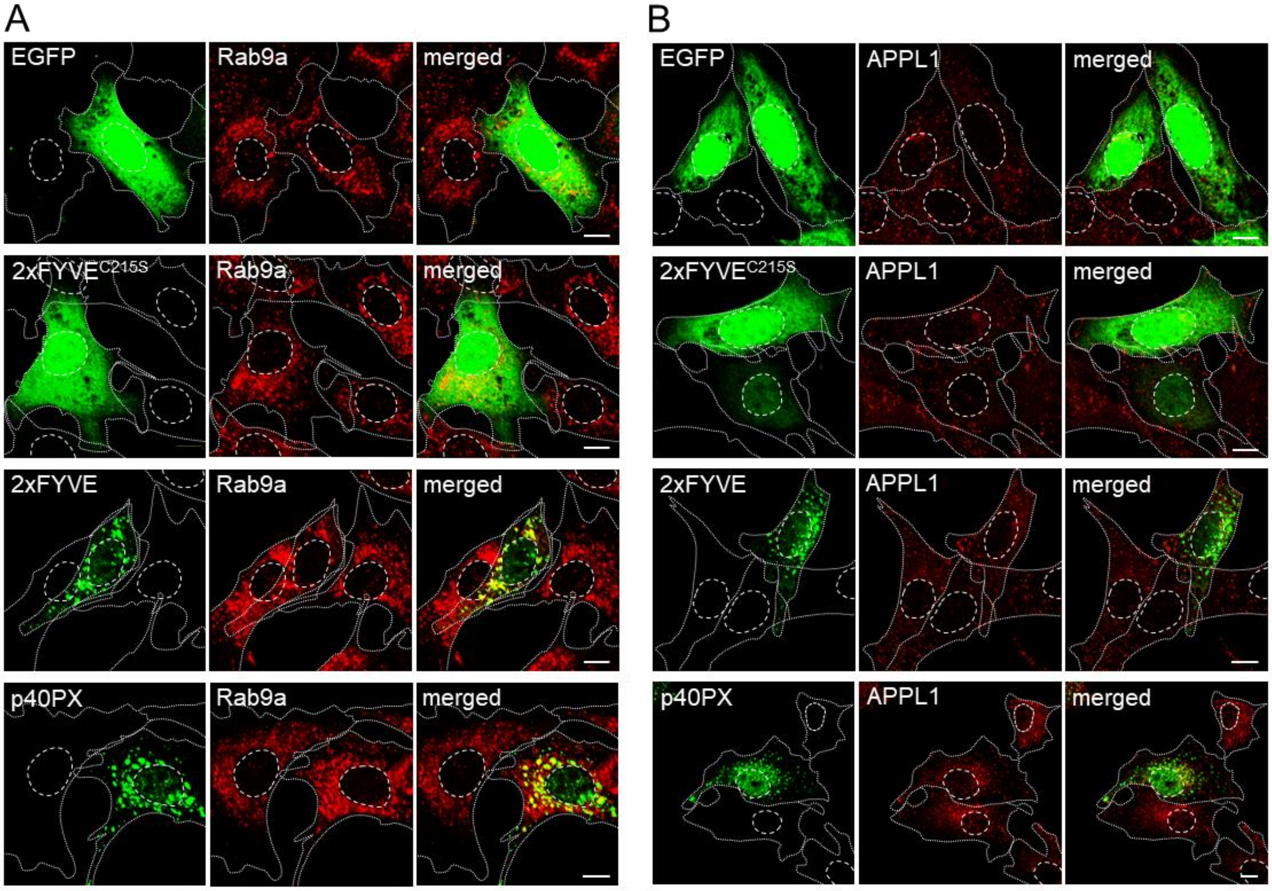

Figure S16. **Colocalization of EGFP-2xFYVE and YFP-PX with Rab9a and APPL1 (***related to Fig. 8***).** (**A-B**) Balb 3T3 cells were transfected either with EGFP-MSCV, EGFP-2xFYVE^{C125S}-MSCV, EGFP-2xFYVE-MSCV, or YFP-p40PX-MSCV for 48 hrs. Fixed and permeabilized cells were stained with rabbit Abs against Rab9a (**A**), Vps35 (**B**), and APPL1 (**C**), followed by the secondary anti-rabbit AF^{594} Abs. Cell borders are indicated by fine dotted lines and nuclei by fine dashed lines. Bars, 10 μm.

Supplementary tables

Table S1. **List of antibody and ligand reagents used in this study**

Target molecule	Reagent	Reference
	**Rab proteins**	
**Rab4**	Rabbit polyclonal (Thermo Fisher Scientific, Cat.No. PA3-912)	Kane et al., 2019
**Rab5a**	Rabbit monoclonal (Cell Signaling, Cat.No. 3547)<br>Mouse monoclonal (Cell Signaling, Cat.No. 46449)	ad sc28570 - Meister et al., 2014<br>ad 46449 - Malik et al., 2019
**Rab7a**	Rabbit monoclonal (Cell Signaling, Cat.No. 9367)	Hong et al., 2015; Hubert et al., 2016
**Rab8a**	Mouse monoclonal IgG_1 (Santa Cruz Biotechnology, Cat.No. sc-81909)	Giacometti et al., 2020
**Rab9a**	Rabbit monoclonal (Cell Signaling, Cat.No. 5118)	Liu et al., 2012
**Rab11a**	Rabbit monoclonal (Cell Signaling, Cat.No. 5589)<br>Mouse monoclonal IgG_1 (Proteintech, Cat.No. 67902-1-Ig)	ad 5589 - Di Matteo et al., 2017
	**Membranous organelle effector proteins and markers**	
**HRS**	Rabbit monoclonal (Cell Signaling, Cat.No. 15087)	Jones et al., 2020
**APPL1**	Rabbit monoclonal (Cell Signaling, Cat.No. 3858)	Jung et al., 2017
**EEA1**	Chicken polyclonal (Invitrogen, Cat.No. 40-5700)<br>Rabbit monoclonal (Cell Signaling, Cat.No. 3288)	ad 40-5700 - Mu et al., 1995; Simonsen et al., 1998; Christoforidis et al., 1999;<br>ad 3288 - Jimenez-Orgaz et al., 2018; Tan et al., 2020
**Lamp1**	Rat monoclonal IgG_{2a} (BD Pharmigen, Cat.No. 553792)	Belaid et al., 2014; Garg et al., 2011
**GM1**	Alexa Fluor CTxB 488 conjugate (Invitrogen, Cat.No. C22841)	Bavari et al., 2002
**TfR (CD71)**	Transferrin Alexa Fluor 594 (Invitrogen, Cat.No. TI3343)<br>Transferrin Alexa Fluor 555 (Invitrogen, Cat.No. T35352)<br>Transferrin Alexa Fluor 488 (Invitrogen, Cat.No. T13342)<br>Monoclonal antibody, clone R17 217.1.3 (rat IgG2a; ATCC TIB 219).	ad TI3343 - Steinert et al., 2008<br>ad T3535 - Razi et al., 2009<br>ad T13342 - Barysch et al., 2010<br>mAb - Mahmutefendić et al., 2011
**GS15**	Mouse monoclonal IgG_1 (BD Biosciences, Cat.No. 610960)	Williams et al., 2014
**GM130**	Mouse monoclonal IgG_1 (BD Biosciences, Cat.No. 610823)	Bagh et al., 2017
**Vti1a**	Mouse monoclonal IgG_1 (BD Biosciences, Cat.No. 611220)	Crawford et al., 2017; Wong and Munro, 2014

**TGN-38**	Sheep polyclonal (Bio-Rad AbD Serotec Limited, Cat.No. AHP499G)	Tenorio et al., 2016
**SNX1**	Mouse monoclonal IgG_1 (Santa Cruz Biotechnology, Cat. No. Sc-376376)	-
**SNX3**	Rabbit polyclonal (Proteintech, Cat.No. 10772-1-AP)	McGough et al., 2018
**SNX4**	Rabbit polyclonal (Invitrogen, Cat.No. PA5-101907)<br>Mouse monoclonal IgG2a (Santa Cruz Biotechnology, Cat. No. sc-271147)	-
**Rab11FIP3**	Rabbit polyclonal (Proteintech, Cat.No. 25843-1-AP)	Walia et al., 2019; Iannantuono and Emeri, 2021
**Rab11FIP5**	Rabbit polyclonal (Proteintech, Cat.No. 14594-1-AP)	He et al., 2018
	**Cytoskeleton**	
**Actin**	Mouse monoclonal (Millipore, Cat.No. MAB1501)	Treda et al., 2016
**Gamma tubulin**	Mouse monoclonal IgG_1 (Invitrogen, Cat.No. MA1-19421)	Simula et al., 2018

Supplementary references:

1. Bagh, M. B., Peng, S., Chandra, G., Zhang, Z., Singh, S. P., Pattabiraman, N., et al. (2017). Misrouting of v-ATPase subunit V0a1 dysregulates lysosomal acidification in a neurodegenerative lysosomal storage disease model. *Nat. Commun.* 8:14612. doi: 10.1038/ncomms14612.
2. Barysch, S. V., Jahn, R., and Rizzoli, S. O. (2010). A fluorescence-based in vitro assay for investigating early endosome dynamics. *Nat. Protoc*. 5, 1127-1137. doi:10.1038/nprot.2010.84.
3. Bavari, S., Bosio, C. M., Wiegand, E., Ruthel, G., Will, A. B., Geisbert, T. W., et al. (2002). Lipid raft microdomains: a gateway for compartmentalized trafficking of Ebola and Marburg viruses. *J. Exp. Med*. 195, 593-602. doi: 10.1084/jem.20011500.
4. Belaid, A., Ndiaye, P. D., Cerezo, M., Cailleteau, L., Brest, P., Klionsky, D. J., et al. (2014). Autophagy and SQSTM1 on the RHOA(d) again: emerging roles of autophagy in the degradation of signaling proteins. *Autophagy* 10, 201-208. doi: 10.4161/auto.27198.
5. Christoforidis, S., McBride, H. M., Burgoyne, R. D., and Zerial, M. (1999). The Rab5 effector EEA1 is a core component of endosome docking. *Nature* 397, 621-625. doi: 10.1038/17618.
6. Crawford, D. C., Ramirez, D. M., Trauterman, B., Monteggia, L. M., and Kavalali, E. T. (2017). Selective molecular impairment of spontaneous neurotransmission modulates synaptic efficacy. *Nat. Commun*. 8, 14436. doi: 10.1038/ncomms14436.
7. Di Matteo, P., Calvello, M., Luin, S., Marchetti, L., and Cattaneo, A. (2017). An optimized procedure for the site-directed labeling of NGF and proNGF for imaging purposes. *Front. Mol. Biosci*. 4, 4. doi: 10.3389/fmolb.2017.00004.
8. Fraser, J., Simpson, J., Fontana, R., Kishi-Itakura, C., Ktistakis, N. T., and Gammoh, N. (2019). Targeting of early endosomes by autophagy facilitates EGFR recycling and signalling. *EMBO Rep.* 20(10), e47734. doi: 10.15252/embr.201947734.
9. Garg, S., Sharma, M., Ung, C., Tuli, A., Barral, D. C., Hava, D. L., et al. (2011). Lysosomal trafficking, antigen presentation, and microbial killing are controlled by the Arf-like GTPase Arl8b. *Immunity* 35, 182-193. doi: 10.1016/j.immuni.2011.06.009.
10. Giacometti, J., Muhvić, D., Grubić-Kezele, T., Nikolić, M., Šoić-Vranić, T., and Bajek, S. (2020). Olive leaf polyphenols (OLPs) stimulate GLUT4 expression and translocation in the skeletal muscle of diabetic rats. *Int J Mol Sci*. 21(23):8981. doi: 10.3390/ijms21238981.
11. He, K., Ma, X., Xu, T., Li, Y., Hodge, A., Zhang, Q., et al. (2018). Axoneme polyglutamylation regulated by Joubert syndrome protein ARL13B controls ciliary targeting of signaling molecules. *Nat Commun*. 9(1):3310. doi: 10.1038/s41467-018-05867-1.
12. Hong, N. H., Qi, A., and Weaver, A. M. (2015). PI(3,5)P2 controls endosomal branched actin dynamics by regulating cortactin-actin interactions. *J. Cell Biol*. 210, 753-769. doi: 10.1083/jcb.201412127.
13. Hubert, V., Peschel, A., Langer, B., Gröger, M., Rees, A., and Kain, R. (2016). LAMP-2 is required for incorporating syntaxin-17 into autophagosomes and for their fusion with lysosomes. *Biol. Open* 5, 1516-1529. doi: 10.1242/bio.018648.
14. Iannantuono, NVG., and Emery, G. (2021). Rab11FIP1 maintains Rab35 at the intercellular bridge to promote actin removal and abscission. *J Cell Sci*. 134(12):jcs244384. doi: 10.1242/jcs.244384.
15. Jimenez-Orgaz, A., Kvainickas, A., Nägele, H., Denner, J., Eimer, S., Dengjel, J., et al. (2018). Control of RAB7 activity and localization through the retromer-TBC1D5 complex enables RAB7-dependent mitophagy. *EMBO J.* 37, 235-254. doi: 10.15252/embj.201797128.

16. Jones, S., King, P.J., Antonescu, C.N., Sugiyama, M.G., Bhamra, A., Surinova, S., et al. (2020). Targeting of EGFR by a combination of antibodies mediates unconventional EGFR trafficking and degradation. *Sci. Rep*. 10(1):663. doi: 10.1038/s41598-019-57153-9.
17. Jones, T., Naslavsky, N., and Caplan, S. (2020). Eps15 Homology Domain Protein 4 (EHD4) is required for Eps15 Homology Domain Protein 1 (EHD1)-mediated endosomal recruitment and fission. *PLoS One* 15(9), e0239657. doi: 10.1371/journal.pone.0239657.
18. Jung, Y. R., Lee, J. H., Sohn, K. C., Lee, Y., Seo, Y. J., Kim, C. D., et al. (2017). Adiponectin signaling regulates lipid production in human sebocytes. *PLoS One* 12, e0169824. doi: 10.1371/journal.pone.0169824.
19. Kane, M. S., Diamonstein, C. J., Hauser, N., Deeken, J. F., Niederhuber, J. E., and Vilboux, T. (2019). Endosomal trafficking defects in patient cells with KIAA1109 biallelic variants. *Genes & Diseases* 6(1), 56–67. doi:10.1016/j.gendis.2018.12.004.
20. Liu, X. D., Ko, S., Xu, Y., Fattah, E. A., Xiang, Q., Jagannath, C., et al. (2012). Transient aggregation of ubiquitinated proteins is a cytosolic unfolded protein response to inflammation and endoplasmic reticulum stress. *J. Biol. Chem*. 287, 19687-19698. doi: 10.1074/jbc.M112.350934.
21. Mahmutefendić, H., Blagojević, G., Ilić Tomaš, M., Kučić, N., and Lučin, P. (2011). Segregation of open Major Histocompatibility Class I conformers at the plasma membrane and during endosomal trafficking reveals conformation-based sorting in the endosomal system. *Int. J. Biochem. Cell Biol*. 43, 504-515 doi: 10.1016/j.biocel.2010.12.002.
22. Malik, N., Nirujogi, R.S., Peltier, J., Macartney, T., Wightman, M., Prescott, A.R., et al. (2019). Phosphoproteomics reveals that the hVPS34 regulated SGK3 kinase specifically phosphorylates endosomal proteins including Syntaxin-7, Syntaxin-12, RFIP4 and WDR44. *Biochem J*. 476, 3081-3107. doi: 10.1042/BCJ20190608.
23. McGough, I.J., de Groot, R.E.A., Jellett, A.P., Betist, M.C., Varandas, K.C., Danson, C.M., et al. (2018). SNX3-retromer requires an evolutionary conserved MON2:DOPEY2:ATP9A complex to mediate Wntless sorting and Wnt secretion. *Nat. Commun*. 9(1):3737. doi: 10.1038/s41467-018-06114-3.
24. Meister, M., Zuk, A., and Tikkanen, R. (2014). Role of dynamin and clathrin in the cellular trafficking of flotillins. *FEBS J*. 281, 2956-2976. doi: 10.1111/febs.12834.
25. Mohan, N., Shen, Y., Dokmanovic, M., Endo, Y., Hirsch, D. S., and Wu, W. J. (2016). VPS34 regulates TSC1/TSC2 heterodimer to mediate RheB and mTORC1/S6K1 activation and cellular transformation. *Oncotarget* 7(32), 52239–52254. doi: 10.18632/oncotarget.10469.
26. Mu, F. T., Callaghan, J. M., Steele-Mortimer, O., Stenmark, H., Parton, R.G., Campbell, P. L., et al. (1995). EEA1, an early endosome-associated protein: EEA1 is a conserved alpha-helical peripheral membrane protein flanked by cysteine "fingers" and contains a calmodulin-binding IQ motif. *J. Biol. Chem*. 270, 13503-13511. doi: 10.15252/embj.201695597.
27. Razi, M., Chan, E. Y., and Tooze, S. A. (2009). Early endosomes and endosomal coatomer are required for autophagy. *J. Cell Biol*. 185, 305-321. doi: 10.1083/jcb.200810098.
28. Simonsen, A., Lippé, R., Christoforidis, S., Gaullier, J. M., Brech, A., Callaghan, J., et al. (1998). EEA1 links PI(3)K function to Rab5 regulation of endosome fusion. *Nature* 394, 494-498. doi: 10.1038/28879.
29. Simula, L., Pacella, I., Colamatteo, A., Procaccini, C., Cancila, V., Bordi, M. et al. (2018). Drp1 Controls Effective T Cell Immune-Surveillance by Regulating T Cell Migration, Proliferation, and cMyc-Dependent Metabolic Reprogramming. *Cell Rep*. 25(11), 3059-3073. doi: 10.1016/j.celrep.2018.11.018.
30. Steinert, S., Lee, E., Tresset, G., Zhang, D., Hortsch, R., Wetzel, R., et al. (2008). A fluorescent glycolipid-binding peptide probe traces cholesterol dependent microdomain-derived trafficking pathways. *PLoS One* 3(8), e2933. doi: 10.1371/journal.pone.0002933.
31. Tan, C.F., Teo, H.S., Park, J.E., Dutta, B., Tse, S.W., Leow, M.K, et al. (2020). Exploring extracellular vesicles biogenesis in hypothalamic cells through a heavy isotope pulse/trace proteomic approach. *Cells* 9(5):1320. doi: 10.3390/cells9051320.
32. Tenorio, M.J., Ross, B.H., Luchsinger, C., Rivera-Dictter, A., Arriagada, C., Acuña, D., et al. (2016). Distinct Biochemical Pools of Golgi Phosphoprotein 3 in the Human Breast Cancer Cell Lines MCF7 and MDA-MB-231. *PLoS One* 28, 11(4):e0154719. doi: 10.1371/journal.pone.0154719.
33. Treda, C., Popeda, M., Ksiazkiewicz, M., Grzela, D. P., Walczak, M. P., Banaszczyk, M., et al. (2016). EGFR activation leads to cell death independent of PI3K/AKT/mTOR in an AD293 cell line. *PLoS One* 11, e0155230. doi: 10.1371/journal.pone.0155230.
34. Walia, V., Cuenca, A., Vetter, M., Insinna, C., Perera, S., Lu, Q., et al. (2019). Akt Regulates a Rab11-Effector Switch Required for Ciliogenesis. *Dev Cell*. 50(2):229-246.e7. doi: 10.1016/j.devcel.2019.05.022.
35. Williams, K. C., McNeilly, R. E., and Coppolino, M. G. (2014). SNAP23, Syntaxin4, and vesicle-associated membrane protein 7 (VAMP7) mediate trafficking of membrane type 1-matrix metalloproteinase (MT1-MMP) during invadopodium formation and tumor cell invasion. *Mol. Biol. Cell* 25, 2061-2070. doi: 10.1091/mbc.E13-10-0582.
36. Wong, M., and Munro, S. (2014). Membrane trafficking. The specificity of vesicle traffic to the Golgi is encoded in the golgin coiled-coil proteins. *Science* 346, 1256898. doi: 10.1126/science.1256898.
37. Yap, C. C., Digilio, L., McMahon, L., and Winckler, B. (2017). The endosomal neuronal proteins Nsg1/NEEP21 and Nsg2/P19 are itinerant, not resident proteins of dendritic endosomes. *Sci. Rep*. 7(1), 10481. doi: 10.1038/s41598-017-07667-x.

Table S2. **Reported subcellular localization and function of used markers**

Marker	Subcellular localization and function
	**Markers of the EE, ERC, TGN, and their activity at EE-ERC-TGN interface**
**APPL1**	*Recruits to membranes of a subset of cortical pre-EEs. Displays the size of a subset of pre-EEs.* Defines a subset of precursor EEs, which are stable sorting stations - often called very early endosomes or pre-EEs. Enables rapid cargo recycling (Kalaidzidis et al., 2015). APPL1 localizes to a subset of Rab5-positive endosomes via interaction with Rab5 and competes with EEA1 for Rab5 binding (Zoncu et al., 2009; Diggins and Webb, 2017).
**Rab4**	*Displays the tubular recycling domain of EEs.* Occupy distinct domain than Rab5 at EEs (Sönnichsen et al., 2000). Reported to be >90% (Marzesco et al., 1998) and almost 100% (Mari et al., 2001) membrane-associated. Activates on EEA1-positive EEs and generates a highly dynamic tubular domain. Activates Arl1, which recruits BIG1 and BIG2 and thereby activates ARF1 and ARF3 to promote assembly of AP-1 and AP-3 and GGA-3 (D'Souza et al., 2014). Constantly nucleates Rab4 domain at Rab5-positive endosomes and generates Rab4-positive tubules that mediate the exit of recycling cargo from EEs (Rink et al., 2005).
**Rab5a**	*Displays the vacuolar domain of EEs.* Rab5-positive endosomes are continuously generated/renewed in the cell periphery and move toward the cell center while growing in size (Rink et al., 2005). Reported to be ~50% in a membrane-associated form (Massignan et al., 2010; Liu and Grant, 2015). Targeted to membranes of EEs by Rabex-5/Rabaptin-5 complexes (Zhu et al., 2009). Activates generation of phosphatidylinositol (PtdIns)3P (PI3P) and generates PI3P-domain at early endosomes. Binds a large number of effector proteins, including EEA1 (required for fusion of endosomes) and Hrs/HGS (required for sorting of ubiquitinated cargo) (Frankel and Audhya, 2018). Excludes RAB-10 and thereby prevents the progression of membranes at the Golgi–endosomal interface, including the progression of EE membranes towards the ERC (Sasidharan et al., 2012).
**Vps34 (PI3 Kinase Class III)**	*Recruits to Rab5-positive EE and Rab7a-positive LE membranes.* Sole class III phosphoinositide 3-kinase that generates PI(3)P (Marat and Haucke, 2016; Schink et al., 2016). In complex with Vps15, Beclin1, and UVRAG targets early endosomes through the interaction between Vps15 and Rab5 (Murray et al., 2002). Through autophagy-specific subunit, ATG14 recruits to contact sites between mitochondria and the ER for localized PI3P production and the formation of autophagosome precursors (Hamasaki et al., 2013).
**SNX1**	*Displays EE membranes* Sorting nexin 1. Enriched on tubular elements of the early endosome membrane (Cozier et al., 2002; Carlton et al., 2004). Binds preferentially to highly curved membranes enriched in PtdIns(3)P or PtdIns(3,5)P_2 through its PX domain. For efficient membrane association, SNX1 also requires a functional BAR domain (Carlton et al., 2004). Colocalizes with SORT1 to tubular endosomal membrane structures called endosome-to-TGN transport carriers (ETCs), which are budding from early endosome vacuoles (Mari et al., 2008). Acts in part as a component of the SNX-BAR retromer. It regulates tubular-based endosome-to-TGN retrieval of the CI-MPR and SORT1 (Carlton et al., 2004; Mari et al., 2008). Regulates the targeting of internalized epidermal growth factor receptors for lysosomal degradation (Cozier et al., 2002).
**SNX3**	*Displays EE membranes.* Sorting nexin 3. Associated with the early endosomes through a single PI3P lipid-binding PX domain. Mediates the transport of anti-TfR from the early to the recycling endosome (Xu et al., 2001; Chen et al., 2013). Interacts with the Vps35 component of retromer (Lucas et al., 2016). SNX3-retromer complex mediates the retrograde endosome-to-TGN transport of WLS (McGough et al., 2018; Harterink et al., 2014). Recruits to phagosomes (Chua et al., 2013).

**EEA1**	*Recruits to the vacuolar domain of EEs and indicates PI3P membrane composition.* Rab5 effector protein present at the subdomain of EEs that acts as a tether for incoming vesicles during homo- and heterotypic fusion of EEs. Defines a subset of EEs (Simonsen et al., 1998; Wilson et al., 2000).
**HRS**	*Recruits to a subset of EEA1-negative peripheral pre-EEs and vacuolar domain of EEs. Displays the PI3P domain of EEs and the site of MVB biogenesis.* ESCRT-0 component that initiates the formation of multivesicular body (MVB) vesicles (McCullough et al., 2013). Localizes in a population of peripheral EEA1-negative endosomes distinct from APPL1-containing endosomes and EEA1-positive endosomes (Flores-Rodriguez et al., 2015). Contains PI3P-binding FYVE domain and localizes at different regions than EEA1 of EEs (Raiborg et al., 2001).
**TfR**	*Traffics the endosomal recycling route (PM-pre-EE-EE-ERC-PM). Displays a significant retention localization within the ERC.* TfR localizes at the PM and after binding of the ligand (Tf) undergo rapid clathrin-dependent endocytosis, internalization into several subsets of pre-EEs, sorting into the tubular domain of EEs and recycling via the fast (Rab4-dependent) and slow (Rab11-dependent) recycling route (Grant and Donaldson 2009; Kalaizidis et al., 2015; Villaseñor et al., 2016; Mahmutefendić et al., 2017).
**Rab11a**	*Displays a subset of REs within the ERC and recycling carriers that tether to PM.* Localizes at the ERC in the pericentriolar region (Sönnichsen et al., 2000) and peripheral recycling vesicles around the cellular tips where it regulates their tethering to PM (Takahashi et al., 2012). In BHK21 cells, almost the entire intracellular pool in a membrane-bound form (Chen et al., 1998); in N2a cells, 65% in a membrane-bound form (Massignan et al., 2010). Functions along the same sequence with ARFs (especially ARF1 and ARF3) at the ERC (Takahashi et al., 2012). Associates with late Golgi/TGN compartments (Goud et al., 2018).
**GM130**	*Resides at membranes of the cis-Golgi.* Cis-Golgi localized member of the Golgin family. Attached to the membrane via GRASP65 (rev by Witkos and Lowe, 2016). Cycles via membranous tubules between the cis-Golgi and late IC stations (Marra et al., 2001).
**GS15**	*Displays transport between medial- and trans-Golgi.* A v-SNARE that complexes with t-SNAREs syntaxin-5, GS28, and Ykt6 (Xu et al., 2002). Mainly found in the medial-Golgi and adjacent tubulo-vesicular elements. Redistributes from the Golgi to the endosomes when the recycling endosome is perturbed, suggesting that GS15 may cycle between endosomes and the Golgi (Tai et al., 2004).
**Vti1a**	*Displays entry sites of EE/RE derived carriers to the TGN.* Found predominantly on the Golgi and TGN (Kreykenbohm et al., 2002). Interacts with STX6 and ST16 to form TGN-localized t-SNARE complex involved in the retrograde transport between EEs/REs and TGN (Mallard et al., 2002). May also form the t-SNARE complex STX5 and STX16 at the cis- and medial-Golgi membranes (Mallard et al., 2002).
**TGN-38**	*Traffic TGN-PM-EE-RE-TGN route. Displays a major retention localization within the TGN.* Integral membrane protein predominantly localized in the TGN. Travels TGN-PM-EE-RE-TGN route and delivered to TGN via REs (Mallet and Maxfield, 1999).
**Rab9a**	*Displays intermediates in the transport between TGN and EEs, and membrane domains of LEs distinct from Rab7-positive domains.* Localizes at LEs and the Golgi. Reported to be ~95% in a membrane-bound form (Seaman et al., 2009). At LEs, defines distinct domain than Rab7 (Barbero et al., 2002). Detected at Rab5-EEs before Rab/Rab7a conversion (Gillingham et al., 2014). Acts downstream of Rab5a and inactivates Rab5a by recruiting Rab5 GAP SGSM3 (Gillingham et al., 2014). It can be activated at TGN and mediate transport to EEs and either transport or conversion from EEs to LEs (Kucera et al., 2016). Recruits Rab36 GAP RUTBC2 and inactivate Rab36 in the endosomal system (Nottingham et al., 2012).

**Rab8a**	*Displays the subset of REs within the ERC and exocytic vesicles derived from the TGN. Indicates tubulation.* Localizes at the ERC (Rahajeng et al., 2012; Vetter et al., 2015), PM (Weekes et al., 2016), late TGN, exocytic vesicles (Grigoriev et al., 2011), and stressed lysosomes (Eguchi et al., 2018). In BHK21 cells, almost the entire intracellular pool is in a membrane-bound form (Chen et al., 1998). It can be activated at Rab11-positive membranes by recruitment of Rabin8 (Rab8-GEF) to Rab11-GTP and mediate the conversion of Rab11-positive membranes to Rab8-positive membranes (Vetter et al., 2015). Rabin8 also recruits Rab8 at ARF6-REs (Homma and Fukuda, 2016). ARF6 regulates Rab8a, and both stimulate the formation of recycling tubules and cell protrusions (Kobayashi and Fukuda, 2012). At late Golgi/TGN compartments, Rab6a mediates recruitment of Rab8a to exocytic vesicles, and Rab8a is required for their fission from the Golgi (Grigoriev et al., 2011; Goud et al., 2018). Mediates Golgi-to-RE transport in polarized MDCK cells (Babbey et al., 2006). Localizes to tubular membranes (Peränen et al., 2011). MICAL-L1 recruits Rab8a to tubular endosomes (Sharma et al., 2009; Giridharan et al., 2012; Kobayashi et al., 2014).
**Rab11FIP3**	*Displays the ERC.* Rab11-Family Interacting Protein 3. Effector of Rab11 GTPases (Horgan and McCaffrey, 2009). Its binding to Rab11a is dependent upon a conserved carboxyl-terminal amphipathic alpha-helix (Prekeris et al., 2003). FIP3 recruits to recycling sorting endosomes by active Rab11 leading to the formation of a heterotetrameric Rab11-FIP3 complex. FIP3 recruits a dynein motor complex that moves cargo to the pericentrosomal ERC (Horgan et al., 2007; Horgan et al., 2010). FIP3 localizes to the ERC and is required for structural integrity and pericentrosomal positioning of the ERC (Horgan et al., 2007; Horgan et al., 2004). It binds to Rab11 and Arf6 simultaneously to form a ternary complex (Fielding et al., 2005).
**Rab11FIP5**	*Displays EEs and tubular recycling endosomes.* Rab11-Family Interacting Protein 5. It can be targeted to membranes independently of Rab11, and Rab11 might be required to stabilize FIP5 association with membranes (Meyers and Prekeris, 2002). FIP5 associates with peripheral tubular recycling endosomes, from where it regulates the sorting of internalized receptors to a slow recycling pathway (Schonteich et al., 2008). FIP5 is known to bind motor protein kinesin II which is required for directing endocytosed proteins into the same recycling pathway (Schonteich et al., 2008). Rab11FIP5 is part of FERARI complex and provides the link to Rab11 (Solinger et al., 2020).
**Lamp1**	*Displays a subset of LE limiting membranes and LE-derived organelles (LRO) and transport carriers.* Mainly present on LEs and lysosomes and at low levels on the PM and EEs (Cook et al., 2004; Janvier and Bonifacino, 2005). Reaches the PM through LRO secretion in a process that requires Rab27a and Gadkin, which recruits AP1 and AP2 (Laulagnier et al., 2011). Present at the early subset of Rab7-positive and Rab7-negative LE membranes and Rab7-positive subset of late LE membranes (Humphries et al., 2011). Present at limiting membranes of LEs. Traffic to lysosomes via the direct route (TGN-EE-LE-Ly) or indirect route (TGN-PM-EE-LE-Ly) (Cook et al., 2004; Lebrand et al., 2002; Janvier and Bonifacino, 2005).
**Rab7a**	*Displays a subset of LE limiting membranes.* Small GTPase that controls transport towards late endosomes and lysosomes (Bucci et al., 2000). Localize at both EEs and LEs. Reported to be ~95% in a membrane-bound form (Seaman et al., 2009). Dispensable for transferring cargo from the LE/MVB to the lysosome and endocytic organelle maintenance (Vanlandingham and Ceresa, 2009). Defines a distinct domain of LEs than Rab9 (Barbero et al., 2002). Recruits retromer on endosomes (Rojas et al., 2008).
**GM1**	*Displays luminal membranes of LEs and PM.* Ganglioside (glycosphingolipid) that can be found at the PM and luminal membranes of LEs (Möbius et al., 1999).

Supplementary references:

1. Backer, J.M. (2008). The regulation and function of Class III PI3Ks: novel roles for Vps34. *Biochem. J.* 410(1), 1-17. doi: 10.1042/BJ20071427.

2. Bago, R., Malik, N., Munson, M. J., Prescott, A. R., Davies, P., Sommer, E., et al. (2014). Characterization of VPS34-IN1, a selective inhibitor of Vps34, reveals that the phosphatidylinositol 3-phosphate-binding SGK3 protein kinase is a downstream target of class III phosphoinositide 3-kinase. *Biochem. J*. 463(3), 413–427. doi:10.1042/BJ20140889.
3. Barbero, P., Bittova, L., and Pfeffer, S. R. (2002). Visualization of Rab9-medicated vesicle transport from endosomes to the trans-Golgi in living cells. *J. Cell Biol*. 156, 511–518. doi: 10.1083/jcb.200109030.
4. Bucci, C., Thomsen, P., Nicoziani, P., McCarthy, J., and van Deurs, B. (2000). Rab7: a key to lysosome biogenesis. *Mol. Biol. Cell* 11, 467-480. doi: 10.1091/mbc.11.2.467.
5. Carlton, J., Bujny, M., Peter, B.J., Oorschot, V.M., Rutherford,. A, Mellor, H., et al. (2004). Sorting nexin-1 mediates tubular endosome-to-TGN transport through coincidence sensing of high- curvature membranes and 3-phosphoinositides. *Curr Biol*. 14(20):1791-800. doi: 10.1016/j.cub.2004.09.077.
6. Chen, C., Garcia-Santos, D., Ishikawa, Y., Seguin, A., Li, L., Fegan, K.H., et al. (2013). Snx3 regulates recycling of the transferrin receptor and iron assimilation. *Cell Metab*. 17(3), 343-52. doi: 10.1016/j.cmet.2013.01.013.
7. Chen, W., Feng, Y., Chen, D., and Wandinger-Ness, A. (1998). Rab11 is required for trans-Golgi network-to-plasma membrane transport and a preferential target for GDP dissociation inhibitor. *Mol. Biol. Cell* 9, 3241-3257. doi: 10.1091/mbc.9.11.3241.
8. Chua, R.Y., and Wong, S.H. (2013). SNX3 recruits to phagosomes and negatively regulates phagocytosis in dendritic cells. *Immunology* 139(1), 30-47. doi: 10.1111/imm.12051.
9. Cook, N. R., Row, P. E, and Davidson, H. W. (2004). Lysosome-associated membrane protein 1 (Lamp1) traffics directly from the TGN to early endosomes. *Traffic* 5, 685-699. doi: 10.1111/j.1600-0854.2004.00212.
10. Cozier, G.E., Carlton, J., McGregor, A.H., Gleeson, P.A., Teasdale, R.D., Mellor, H., and Cullen, P.J. (2002). The phox homology (PX) domain-dependent, 3-phosphoinositide-mediated association of sorting nexin-1 with an early sorting endosomal compartment is required for its ability to regulate epidermal growth factor receptor degradation. *J Biol Chem*. 277(50):48730-6. doi: 10.1074/jbc.M206986200.
11. Diggins, N. L., and Webb, D. J. (2017). APPL1 is a multifunctional endosomal signaling adaptor protein. *Biochem. Soc. Trans*. 45, 771-779. doi: 10.1042/BST20160191.
12. D'Souza, R. S., Semus, R., Billings, E. A., Meyer, C. B., Conger, K., and Casanova, J. E. (2014). Rab4 orchestrates a small GTPase cascade for recruitment of adaptor proteins to early endosomes. *Curr. Biol*. 24, 1187-1198. doi: 10.1016/j.cub.2014.04.003.
13. Dutta, D., and Donaldson, J. G. (2015). Sorting of clathrin-independent cargo proteins depends on Rab35 delivered by clathrin-mediated endocytosis. *Traffic* 16, 994-1009. doi: 10.1111/tra.12302.
14. Eguchi, T., Kuwahara, T., Sakurai, M., Komori, T., Fujimoto, T., Ito, G., et al. (2018). LRRK2 and its substrate Rab GTPases are sequentially targeted onto stressed lysosomes and maintain their homeostasis. *Proc. Natl. Acad. Sci. USA* 115, E9115-E9124. doi: 10.1073/pnas.1812196115.
15. Fielding, A. B., Schonteich, E., Matheson, J., Wilson, G., Yu, X., Hickson, G. R., et al. (2005). Rab11-FIP3 and FIP4 interact with Arf6 and the exocyst to control membrane traffic in cytokinesis. *The EMBO journal*, 24(19), 3389–3399. doi:10.1038/sj.emboj.7600803.
16. Flores-Rodriguez, N., Kenwright, D. A., Chung, P. H., Harrison, A. W., Stefani, F., Waigh, T. A., et al. (2015). ESCRT-0 marks an APPL1-independent transit route for EGFR between the cell surface and the EEA1-positive early endosome. *J. Cell Sci*. 128, 755-767. doi: 10.1242/jcs.161786.
17. Frankel, E. B., and Audhya, A. (2018). ESCRT-dependent cargo sorting at multivesicular endosomes. *Semin. Cell Dev. Biol*. 74, 4-10. doi:10.1016/j.semcdb.2017.08.020.
18. Gillingham, A. K., Sinka, R., Torres, I. L., Lilley, K. S., and Munro, S. (2014). Toward a comprehensive map of the effectors of Rab GTPases. *Dev. Cell* 31, 358-373. doi:10.1016/j.devcel.2014.10.007.
19. Giridharan, S. S., Cai, B., Naslavsky, N., and Caplan, S. (2012). Trafficking cascades mediated by Rab35 and its membrane hub effector, MICAL-L1. Commun. *Integr. Biol*. 5, 384-387. doi: 10.4161/cib.20064.
20. Grant, B. D., and Donaldson, J. G. (2009). Pathways and mechanisms of endocytic recycling. *Nat. Rev. Mol. Cell Biol*. 10, 597–608. doi: 10.1038/nrm2755.
21. Hamasaki, M., Furuta, N., Matsuda, A., Nezu, A., Yamamoto, A., Fujita, N., et al. (2013). Autophagosomes form at ER-mitochondria contact sites. *Nature* 495(7441), 389-93. doi: 10.1038/nature11910.
22. Harterink, M., Port, F., Lorenowicz, M.J., McGough, I.J., Silhankova, M., Betist, M.C., et al. (2011). A SNX3-dependent retromer pathway mediates retrograde transport of the Wnt sorting receptor Wntless and is required for Wnt secretion. *Nat. Cell Biol*. 13(8), 914-923. doi: 10.1038/ncb2281.
23. Homma, Y., and Fukuda, M. (2016). Rabin8 regulates neurite outgrowth in both GEF activity-dependent and -independent manners. *Mol. Biol. Cell* 27, 2107-2118. doi: 10.1091/mbc.E16-02-0091.
24. Horgan, C.P., Walsh, M., Zurawski, T.H., and McCaffrey, M.W. (2004). Rab11-FIP3 localises to a Rab11-positive pericentrosomal compartment during interphase and to the cleavage furrow during cytokinesis. *Biochem Biophys Res Commun*. 319(1):83-94. doi: 10.1016/j.bbrc.2004.04.157.
25. Horgan, C.P., Oleksy, A., Zhdanov, A.V., Lall, P.Y., White, I.J., Khan, A.R., et al. (2007). Rab11-FIP3 is critical for the structural integrity of the endosomal recycling compartment. *Traffic* 8(4):414-30. doi: 10.1111/j.1600-0854.2007.00543.x.
26. Horgan, C.P., and McCaffrey, M.W. (2009). The dynamic Rab11-FIPs. *Biochem Soc Trans*. 37(Pt 5):1032-6. doi: 10.1042/BST0371032.
27. Horgan, C.P., Hanscom, S.R., Jolly, R.S., Futter, C.E., and McCaffrey, M.W. (2010). Rab11-FIP3 links the Rab11 GTPase and cytoplasmic dynein to mediate transport to the endosomal-recycling compartment. *J Cell Sci*. 123(Pt 2):181-91. doi: 10.1242/jcs.052670.
28. Humphries, W. H., Szymanski, C. J., and Payne, C. K. (2011). Endo-lysosomal vesicles positive for Rab7 and LAMP1 are terminal

vesicles for the transport of dextran. *PLoS One* 6, e26626. doi: 10.1371/journal.pone.0026626.
29. Janvier, K., and Bonifacino, J. S. (2005). Role of the endocytic machinery in the sorting of lysosome-associated membrane proteins. *Mol. Biol. Cell* 16, 4231-4242. doi: 10.1091/mbc.e05-03-0213.
30. Kalaidzidis, I., Miaczynska, M., Brewinska-Olchowik, M., Hupalowska, A., Ferguson, C., Parton, R. G., et al. (2015). APPL endosomes are not obligatory endocytic intermediates but act as stable cargo-sorting compartments. *J. Cell Biol*. 211, 123–144. doi: 10.1083/jcb.201311117.
31. Kobayashi, T., Deak, M., Morrice, N., and Cohen, P. (1999). Characterization of the structure and regulation of two novel isoforms of serum- and glucocorticoid-induced protein kinase. *Biochem J*. 15;344, 189-197. doi: 10.1042/0264-6021:3440189.
32. Kreykenbohm, V., Wenzel, D., Antonin, W., Atlachkine, V., and Von Mollard, G. F. (2002). The SNAREs vti1a and vti1b have distinct localization and SNARE complex partners. *Eur. J. Cell Biol*. 81,273-280. doi: 10.1078/0171-9335-00247.
33. Kucera, A., Borg Distefano, M., Berg-Larsen, A., Skjeldal, F., Repnik, U., Bakke, O., et al. (2016). Spatiotemporal resolution of Rab9 and CI-MPR dynamics in the endocytic pathway. *Traffic* 17, 211-229. doi: 10.1111/tra.12357.
34. Laulagnier, K., Schieber, N. L., Maritzen, T., Haucke, V., Parton, R. G., and Gruenberg, J. (2011). Role of AP1 and Gadkin in the traffic of secretory endo-lysosomes. *Mol. Biol. Cell* 22, 2068-2082. doi: 10.1091/mbc.E11-03-0193.
35. Lebrand, C., Corti, M., Goodson, H., Cosson, P., Cavalli, V., Mayran, N., et al. (2002). Late endosome motility depends on lipids via the small GTPase Rab7. *EMBO J*. 21, 1289-1300. doi: 10.1093/emboj/21.6.1289.
36. Lucas, M., Gershlick, D. C., Vidaurrazaga, A., Rojas, A. L., Bonifacino, J. S., and Hierro, A. (2016). Structural Mechanism for Cargo Recognition by the Retromer Complex. *Cell* 167(6), 1623–1635.e14. doi:10.1016/j.cell.2016.10.056.
37. Mahmutefendić, H., Blagojević Zagorac, G., Grabušić, K., Karleuša, Lj., Maćešić, S., Momburg, F., and Lučin, P. (2017). Late endosomal recycling of open MHC-I conformers. *J. Cell. Physiol*. 232, 872-887. doi: 10.1002/jcp.25495.
38. Mahmutefendić, H., Blagojević, G., Tomaš, M. I., Kučić, N., and Lučin, P. (2011). Segregation of open Major Histocompatibility Class I conformers at the plasma membrane and during endosomal trafficking reveals conformation-based sorting in the endosomal system. *Int. J. Biochem. Cell Biol*. 43, 504-515. doi:10.1016/j.biocel.2010.12.002.
39. Malik, N., Macartney, T., Hornberger, A., Anderson, K. E., Tovell, H., Prescott, A. R., et al. (2018). Mechanism of activation of SGK3 by growth factors via the Class 1 and Class 3 PI3Ks. *Biochem. J*. 2;475(1), 117-135. doi: 10.1042/BCJ20170650.
40. Mallard, F., Tang, B. L., Galli, T., Tenza, D., Saint-Pol, A., Yue, X., et al. (2002). Early/recycling endosomes-to TGN transport involves two SNARE complexes and a Rab6 isoform. *J. Cell Biol*. 156, 653-664. doi: 10.1083/jcb.200110081.
41. Mallet, W. G., and Maxfield, F. R. (1999). Chimeric forms of furin and TGN38 are transported with the plasma membrane in the trans-Golgi network via distinct endosomal pathways. *J. Cell Biol*. 146, 345-359. doi: 10.1083/jcb.146.2.345.
42. Marat, A. L., and Haucke, V. (2016). Phosphatidylinositol 3-phosphates—at the interface between cell signalling and membrane traffic. EMBO J. 35, 561–579. doi:10.15252/embj.201593564.
43. Mari, M., Macia, E., Le Marchand-Brustel, Y., and Cormont, M. (2001). Role of the FYVE finger and the RUN domain for the subcellular localization of Rabip4. *J. Biol. Chem*. 276, 42501-42508. doi: 10.1074/jbc.M104885200.
44. Mari, M., Bujny, M.V., Zeuschner, D., Geerts, W.J., Griffith, J., Petersen, C.M., et al. (2008). SNX1 defines an early endosomal recycling exit for sortilin and mannose 6-phosphate receptors. *Traffic*. 9(3):380-93. doi: 10.1111/j.1600-0854.2007.00686.x.
45. Marra, P., Maffucci, T., Daniele, T., Tullio, G. D., Ikehara, Y., Chan, E. K., et al. (2001). The GM130 and GRASP65 Golgi proteins cycle through and define a subdomain of the intermediate compartment. *Nat. Cell Biol*. 3, 1101-1113. doi: 10.1038/ncb1201-1101.
46. Massignan, T., Biasini, E., Lauranzano, E., Veglianese, P., Pignataro, M., Fioriti, L., et al. (2010). Mutant prion protein expression is associated with an alteration of the Rab GDP dissociation inhibitor alpha (GDI)/Rab11 pathway. *Mol. Cell. Proteom.* 9, 611-622. doi: 10.1074/mcp.M900271-MCP200.
47. McCullough, J., Colf, L. A., and Sundquist, W. I. (2013). Membrane fission reactions of the mammalian ESCRT pathway. *Annu. Rev. Biochem*. 82, 663-692. doi: 10.1146/annurev-biochem-072909-101058.
48. McGough, I.J., de Groot, R.E.A., Jellett, A.P., Betist, M.C., Varandas, K.C., Danson, C.M., et al. (2018). SNX3-retromer requires an evolutionary conserved MON2:DOPEY2:ATP9A complex to mediate Wntless sorting and Wnt secretion. *Nat. Commun*. 9(1):3737. doi: 10.1038/s41467-018-06114-3.
49. Meyers, J.M., and Prekeris, R. (2002). Formation of mutually exclusive Rab11 complexes with members of the family of Rab11-interacting proteins regulates Rab11 endocytic targeting and function. *J Biol Chem*. 277(50):49003-10. doi: 10.1074/jbc.M205728200.
50. Möbius, W., Herzog, V., Sandhoff, K., and Schwarzmann, G. (1999). Intracellular distribution of a biotin-labeled ganglioside, GM1, by immunoelectron microscopy after endocytosis in fibroblasts. *J. Histochem. Cytochem*. 47, 1005-1014. doi: 10.1177/002215549904700804.
51. Murray, J. T., Panaretou, C., Stenmark, H., Miaczynska, M., and Backer, J. M. (2002). Role of Rab5 in the recruitment of hVps34/p150 to the early endosome. *Traffic* 3(6), 416-27. doi: 10.1034/j.1600-0854.2002.30605.x.
52. Nottingham, R. M., Pusapati, G. V., Ganley, I. G., Barr, F. A., Lambright, D. G., and Pfeffer, S. R. (2012). RUTBC2 protein, a Rab9A effector and GTPase-activating protein for Rab36. *J. Biol. Chem*. 287, 22740-22748. doi: 10.1074/jbc.M112.362558.
53. Peränen, J. (2011). Rab8 GTPase as a regulator of cell shape. *Cytoskeleton* (Hoboken). 68, 527-539. doi:10.1002/cm.20529.
54. Prekeris, R. (2003). Rabs, Rips, FIPs, and endocytic membrane traffic. *ScientificWorldJournal*. 15;3:870-80. doi: 10.1100/tsw.2003.69.
55. Rahajeng, J., Panapakkam Giridharan, S. S., Cai, B., Naslavsky, N., Caplan, S. (2012). MICAL-L1 is a tubular endosomal membrane hub that connects Rab35 and ARF6 with Rab8a. *Traffic* 13, 82-93. doi: 10.1111/j.1600-0854.2011.01294.x.
56. Raiborg, C., Bache, K. G., Mehlum, A., and Stenmark, H. (2001). Function of Hrs in endocytic trafficking and signalling. *Biochem. Soc. Trans*. 29, 472-475. doi: 10.1042/bst0290472.
57. Rink, J., Ghigo, E., Kalaidzidis, Y., and Zerial, M. (2005). Rab conversion as a mechanism of progression from early to late

endosomes. *Cell* 122, 735–749. doi: 10.1016/j.cell.2005.06.043.

58. Rojas, R., van Vlijmen, T., Mardones, G. A., Prabhu, Y., Rojas, A. L., Mohammed, S., et al. (2008). Regulation of retromer recruitment to endosomes by sequential action of Rab5 and Rab7. *J. Cell Biol*. 183, 513-526. doi: 10.1083/jcb.200804048.
59. Sasidharan, N., Sumakovic, M., Hannemann, M., Hegermann, J., Liewald, J. F., Olendrowitz, C., et al. (2012). RAB-5 and RAB-10 cooperate to regulate neuropeptide release in Caenorhabditis elegans. *Proc. Natl. Acad. Sci*. USA 109, 18944-18949. doi: 10.1073/pnas.1203306109.
60. Schink, K. O., Tan, K.-W., and Stenmark, H. (2016). Phosphoinositides in Control of Membrane Dynamics. *Annu. Rev. Cell Dev. Biol.* 32, 143–171. doi:10.1146/annurev-cellbio-111315-125349.
61. Schöneberg, J., Lee, I. H., Iwasa, J. H., and Hurley, J. H. (2017). Reverse-topology membrane scission by the ESCRT proteins. *Nat. Rev. Mol. Cell Biol*. 18, 5-17. doi: 10.1038/nrm.2016.121.
62. Schonteich, E., Wilson, G.M., Burden, J., Hopkins, C.R., Anderson, K., Goldenring, J.R., and Prekeris, R. (2008). The Rip11/Rab11-FIP5 and kinesin II complex regulates endocytic protein recycling. *J Cell Sci*. 121(Pt 22):3824-33. doi: 10.1242/jcs.032441.
63. Seaman, M. N., Harbour, M. E., Tattersall, D., Read, E., and Bright, N. (2009). Membrane recruitment of the cargo-selective retromer subcomplex is catalysed by the small GTPase Rab7 and inhibited by the Rab-GAP TBC1D5. *J. Cell Sci.* 122, 2371-2382. doi: 10.1242/jcs.048686.
64. Sharma, M., Giridharan, S. S., Rahajeng, J., Naslavsky, N., and Caplan, S. (2009). MICAL-L1 links EHD1 to tubular recycling endosomes and regulates receptor recycling. *Mol. Biol. Cell* 20, 5181-5194. doi: 10.1091/mbc.e09-06-0535.
65. Simonsen, A., Lippe, R., Christoforidis, S., Gaullier, J. M., Brech, A., Callaghan, J., et al. (1998). EEA1 links PI(3)K function to Rab5 regulation of endosome fusion. *Nature* 394, 494-498. doi: 10.1038/28879.
66. Solinger, J.A., Rashid, H.O., Prescianotto-Baschong, C., and Spang, A.. (2020). FERARI is required for Rab11-dependent endocytic recycling. *Nat Cell Biol*. 22(2):213-224. doi: 10.1038/s41556-019-0456-5.
67. Sönnichsen, B., De Renzis, S., Nielsen, E., Rietdorf, J., and Zerial, M. (2000). Distinct membrane domains on endosomes in the recycling pathway visualized by multicolor imaging of Rab4, Rab5, and Rab11. *J. Cell Biol*. 149, 901–914. doi: 10.1083/jcb.149.4.901.
68. Tai, G., Lu, L., Wang, T. L., Tang, B. L., Goud, B., Johannes L., et al. (2004). Participation of the syntaxin 5/Ykt6/GS28/GS15 SNARE complex in transport from the early/recycling endosome to the trans-Golgi network. *Mol. Biol. Cell* 15, 4011-4022. doi: 10.1091/mbc.e03-12-0876.
69. Vanlandingham, P. A., and Ceresa, B. P. (2009). Rab7 regulates late endocytic trafficking downstream of multivesicular body biogenesis and cargo sequestration. *J. Biol. Chem*. 284, 12110–12124. doi: 10.1074/jbc.M809277200.
70. Vetter, M., Stehle, R., Basquin, C., Lorentzen, E. (2015). Structure of Rab11-FIP3-Rabin8 reveals simultaneous binding of FIP3 and Rabin8 effectors to Rab11. *Nat. Struct. Mol. Biol*. 22, 695-702. doi: 10.1038/nsmb.3065.
71. Villaseñor, R., Kalaidzidis, Y., and Zerial, M. (2016). Signal processing by the endosomal system. *Curr. Opin. Cell Biol*. 39:53-60. doi: 10.1016/j.ceb.2016.02.002.
72. Weekes, M. P., Tomasec, P., Huttlin, E. L., Fielding, C. A., Nusinow, D., Stanton, R. J., et al. (2014). Quantitative temporal viromics: an approach to investigate hostpathogen interaction. *Cell* 157, 1460–1472. doi: 10.1016/j.cell.2014.04.028.
73. Wilson, J. M., de Hoop, M., Zorzi, N., Toh, B. H., Dotti, C. G., and Parton, R. G. (2000). EEA1, a tethering protein of the early sorting endosome, shows a polarized distribution in hippocampal neurons, epithelial cells, and fibroblasts. *Mol. Biol. Cell* 11, 2657-2671. doi: 10.1091/mbc.11.8.2657.
74. Xu, Y., Hortsman, H., Seet, L., Wong, S.H., and Hong, W. (2001). SNX3 regulates endosomal function through its PX-domain-mediated interaction with PtdIns(3)P. *Nat. Cell Biol.* 3(7), 658-66. doi: 10.1038/35083051.
75. Xu, Y., Martin, S., James, D. E., and Hong, W. (2002). GS15 forms a SNARE complex with syntaxin 5, GS28, and Ykt6 and is implicated in traffic in the early cisternae of the Golgi apparatus. *Mol. Biol. Cell* 13, 3493-3507. doi: 10.1091/mbc.e02-01-0004.
76. Zoncu, R., Perera, R. M., Balkin, D. M., Pirruccello, M., Toomre, D., and De Camilli, P. (2009). A phosphoinositide switch controls the maturation and signaling properties of APPL endosomes. *Cell* 136, 1110–1121. doi: 10.1016/j.cell.2009.01.032.
